# Exercise Interventions for PTSD: A Systematic Review and Network Meta-Analysis of Overall Effects, Comparative Effectiveness, and Ranking Uncertainty

**DOI:** 10.3390/healthcare14193166

**Published:** 2026-09-24

**Authors:** Hong-Bum Eun, Sang-Keon Yoo

**Affiliations:** 1 Major in Exercise & Sports Science, Department of Beauty Health Design, Open Cyber University of Korea, Seoul 02087, Republic of Korea; 2 Department of Sport ICT Convergence, The Graduate School, Sangmyung University, Seoul 03016, Republic of Korea

**Keywords:** post-traumatic stress disorder, PTSD, exercise, physical activity, network meta-analysis, treatment ranking, SUCRA, CINeMA, systematic review

## Abstract

**Highlights:**

**What are the main findings?**

- Exercise and movement-based interventions were associated with a moderate average reduction in PTSD symptoms, although the prediction interval indicated variability in effects across future settings.
- Treatment rankings were not robust: resistance exercise had the highest probability of ranking first, whereas yoga had the most favorable SUCRA and mean rank, and rankings changed when the intervention definition was broadened.

**What are the implications of the main findings?**

- Exercise may be considered as an adjunctive component of PTSD care, but treatment rankings alone should not determine which exercise modality is selected.
- Exercise selection should prioritize patient preference, clinical suitability, safety, feasibility, accessibility, and sustainability until stronger head-to-head evidence becomes available.

**Abstract:**

**Background/Objectives:** Exercise has been investigated as an adjunctive intervention for post-traumatic stress disorder (PTSD), but the comparative effectiveness of exercise modalities remains uncertain. This study evaluated the effects of exercise and movement-based interventions and whether the available evidence supports a reliable hierarchy of exercise modalities. **Methods:** PubMed, APA PsycINFO, the Cochrane Central Register of Controlled Trials (CENTRAL), and ScienceDirect were searched through 31 January 2026 for randomized controlled trials in adults with PTSD or clinically significant symptoms. Risk of bias was assessed using the Cochrane Risk of Bias 2 (RoB 2) tool, and network confidence using Confidence in Network Meta-Analysis (CINeMA). Post-treatment PTSD severity was synthesized using random-effects meta-analysis with restricted maximum likelihood and Knapp–Hartung adjustment. A frequentist network meta-analysis used the strict exercise network as the principal comparative framework, with a broad exercise and movement-based network evaluated as a sensitivity analysis. **Results:** Twenty studies were included. Fifteen trials (863 participants) contributed to the meta-analysis, showing reduced PTSD symptoms (Hedges’ *g* = −0.49, 95% confidence interval (CI) −0.69 to −0.28; I^2^ = 40.2%), although the 95% prediction interval crossed the null (−1.01 to 0.04). Eleven trials meeting the strict physical-exercise definition and providing compatible raw post-treatment data showed a similar effect (*g* = −0.44, 95% CI −0.66 to −0.20). The strict network included five nodes and 754 participants. No exercise modality showed clear superiority over active control, and no head-to-head comparisons between exercise modalities were available. Resistance exercise had the highest probability of ranking first, whereas yoga had the highest surface under the cumulative ranking curve (SUCRA) value (0.809); confidence in all network comparisons was very low. **Conclusions:** Exercise and movement-based interventions may reduce PTSD symptoms, but current evidence does not support a reliable hierarchy of exercise modalities. Rankings should not be interpreted as clinical superiority without direct comparative evidence.

## 1. Introduction

Post-traumatic stress disorder (PTSD) is a clinically disabling condition that may develop following exposure to traumatic events and is characterized by persistent intrusion symptoms, avoidance, negative alterations in cognition and mood, and heightened arousal and reactivity [1]. In addition to its core psychological symptoms, PTSD is frequently associated with substantial functional impairment and a complex burden of physical and mental health problems [2,3]. Current clinical guidelines emphasize trauma-focused psychotherapies, including Cognitive Processing Therapy, Prolonged Exposure, and Eye Movement Desensitization and Reprocessing, as established treatments for PTSD, while pharmacological treatments are also used when clinically indicated [4]. Nevertheless, variability in treatment response, patient preferences, barriers to treatment access, and the broader physical-health burden associated with PTSD have contributed to continued interest in complementary and adjunctive therapeutic approaches [3,4,5].

Exercise has increasingly been investigated as one such approach [6,7]. Structured physical activity may be particularly relevant to PTSD care because it can be delivered in different formats and settings and may complement established psychological or pharmacological treatments without necessarily replacing trauma-focused care [6,7]. Randomized trials have evaluated a wide range of interventions, including aerobic exercise, resistance exercise, yoga, and multimodal exercise, as well as interventions incorporating stretching, breathing, meditation, or other movement-based components [8,9]. As this evidence base has expanded, systematic reviews and meta-analyses have generally suggested that exercise or physical-activity interventions can reduce PTSD symptom severity, although estimates of effect magnitude and conclusions regarding specific intervention types have varied [6,7,10].

Recent quantitative syntheses illustrate this uncertainty. A recent systematic review and meta-analysis of randomized trials reported an overall beneficial effect of exercise on PTSD symptoms and observed favorable subgroup estimates for yoga and resistance training [11]. A 2025 network meta-analysis examining exercise type and dose likewise supported an overall beneficial effect of exercise and ranked multicomponent exercise as the most favorable modality, while also proposing potentially optimal exercise-dose characteristics [12]. These findings are clinically relevant because they move the research question beyond whether exercise is effective toward the more consequential question of which type of exercise should be selected. However, differences observed across modality-specific subgroups do not necessarily establish comparative superiority, because such estimates may arise from different sets of trials rather than randomized head-to-head comparisons [13,14]. Similarly, probabilistic rankings generated by network meta-analysis may appear decisive even when the underlying relative-effect estimates are sparse or imprecise [15,16,17].

This distinction is particularly important in the literature on exercise interventions for PTSD. Previous trials have varied not only in exercise modality but also in how exercise interventions were defined, participant eligibility criteria, trauma characteristics, exercise dose, comparator intensity, concomitant treatment, and PTSD outcome-assessment methods [7,11,12]. Relative effects obtained against waitlist or no-treatment controls may differ from those obtained against structured educational, attention, psychological, or other active control conditions [18]. Similarly, effect estimates may also vary according to whether PTSD symptoms are assessed using clinician-administered or self-reported measures [19]. In addition, interventions broadly categorized as exercise or mind–body treatments may differ substantially in the extent to which structured physical activity constitutes the primary therapeutic component [20,21]. Consequently, combining clinically heterogeneous interventions within a single network may increase statistical connectivity while simultaneously reducing the plausibility of the transitivity assumption [15].

These concerns become especially important when network meta-analysis is used to establish treatment rankings. Network meta-analysis combines direct and indirect evidence to estimate relative effects across multiple interventions and can generate probabilistic treatment rankings [16]. However, rankings are secondary summaries of the underlying relative-effect estimates and do not independently establish clinical superiority [15,16]. Methodological guidance cautions against interpreting the probability of being best or related ranking measures in isolation, particularly when direct evidence is sparse, estimates are imprecise, or heterogeneity and incoherence are present [15,16]. Moreover, rankings are conditional on the treatments and node definitions included in the network; changes in intervention classification may therefore alter the apparent hierarchy without strengthening the underlying comparative evidence [22,23].

Evaluation of exercise interventions for PTSD should therefore distinguish at least two related but different questions. The first is whether exercise and movement-based interventions reduce PTSD symptoms on average compared with control conditions. The second, and more demanding, question is whether the available randomized evidence is sufficiently connected, coherent, precise, and credible to conclude that one exercise modality is superior to another. Addressing the latter question requires consideration not only of treatment rankings but also of direct and indirect evidence, network geometry, transitivity, heterogeneity, inconsistency, risk of bias, and confidence in individual network estimates [15,24].

A further methodological issue concerns the operational definition of exercise. Exercise is conventionally distinguished from broader physical activity as planned, structured, and repetitive activity undertaken to improve or maintain physical fitness [20]. A strict network based on this concept may provide greater clinical coherence but may also reduce the number of eligible trials and treatment nodes. Conversely, a broader analytical framework incorporating breathing, stretching, meditation, or other movement-based interventions may increase the amount of available evidence while introducing greater clinical heterogeneity. Decisions about whether clinically similar interventions should be combined within or separated across treatment nodes are consequential in network meta-analysis and may influence network geometry, heterogeneity, inconsistency, and treatment rankings [23,25]. Comparing strict and broad analytical frameworks therefore provides an opportunity to evaluate whether conclusions regarding relative effectiveness and treatment ranking are robust to intervention classification or are partly determined by how the network itself is constructed. The present review differs from previous quantitative syntheses in several respects. Rather than treating all exercise- and movement-related interventions as a single comparative framework, we prespecified a strict exercise network and examined a broad exercise and movement-based network separately as a sensitivity analysis. We also evaluated whether treatment rankings were robust to node definition, assessed confidence in network estimates using the Confidence in Network Meta-Analysis (CINeMA) framework [24], and maintained a common raw immediate post-treatment estimand for the primary quantitative synthesis. Thus, the principal contribution of the present study is not the generation of an additional treatment ranking, but the evaluation of whether the available randomized evidence is sufficiently robust to support such a ranking.

Against this background, the present systematic review and meta-analysis had two complementary objectives. First, we estimated the average effect of exercise and movement-based interventions on immediate post-treatment PTSD symptom severity using a conventional random-effects meta-analysis [26] and examined the robustness of this finding. Second, we evaluated the comparative effects of eligible exercise modalities using network meta-analysis and examined whether the available evidence supports a sufficiently reliable treatment hierarchy to inform clinical decision-making.

## 2. Materials and Methods

### 2.1. Study Design and Reporting Guidelines

This systematic review and meta-analysis evaluated randomized controlled trials of exercise and movement-based interventions for adults with post-traumatic stress disorder (PTSD). The review was conducted and reported in accordance with the Preferred Reporting Items for Systematic Reviews and Meta-Analyses 2020 statement (PRISMA 2020) and the PRISMA extension for network meta-analyses (PRISMA-NMA). The review protocol was registered in the International Prospective Register of Systematic Reviews (PROSPERO; registration number: CRD420261323629) and is available at: https://www.crd.york.ac.uk/PROSPERO/view/CRD420261323629 (accessed on 19 September 2026).

Following registration, the analytical framework was refined to distinguish the strict exercise network from the broad exercise and movement-based sensitivity network and to incorporate additional assessments of ranking uncertainty and network inconsistency. These refinements were implemented after study selection and intervention coding but before final network model fitting and treatment-ranking analyses.

### 2.2. Search Strategy

Electronic searches were conducted in PubMed, APA PsycINFO, the Cochrane Central Register of Controlled Trials (CENTRAL), and ScienceDirect from database inception through 31 January 2026. The final database searches were conducted on 7 February 2026. Records available through 31 January 2026 were eligible for inclusion.

Search strategies were constructed using database-specific Boolean combinations rather than isolated search terms. For PubMed, the PTSD concept (“Stress Disorders, Post-Traumatic” [Mesh] OR “post-traumatic stress disorder” [Title/Abstract] OR “posttraumatic stress disorder” [Title/Abstract] OR PTSD [Title/Abstract]) was combined using AND with an exercise/movement concept incorporating controlled vocabulary and free-text terms, and subsequently with a randomized-trial concept (randomized controlled trial [Publication Type] OR controlled clinical trial [Publication Type] OR random* [Title/Abstract] OR trial [Title/Abstract]). For APA PsycINFO, title and abstract terms for PTSD were combined using AND with corresponding exercise/movement terms and a randomized/controlled-trial block. For CENTRAL, the PTSD block (#3) was combined using AND with the exercise/movement block (#10), yielding the final search #11 (#3 AND #10); no additional randomized-trial filter was applied because CENTRAL is a trials-focused database. Because ScienceDirect restricts the number of Boolean operators permitted within a single search field, four complementary searches were conducted, each combining a PTSD block, an intervention block, and a randomized-trial block using AND. The complete database-specific Boolean search strings, including all OR combinations, controlled vocabulary, field tags, and platform-specific syntax, are provided in Supplementary Table S1.

Reference lists of eligible randomized trials and relevant systematic reviews were manually screened to identify additional potentially eligible reports. When multiple publications appeared to originate from the same randomized cohort, author lists, recruitment setting, participant characteristics, sample size, intervention and comparator groups, intervention duration, outcome measures, and trial-registration information were compared. Reports judged to represent the same randomized cohort were linked under a common study identifier to avoid double counting.

### 2.3. Eligibility Criteria

Eligibility criteria were prespecified according to the Participants, Interventions, Comparators, Outcomes, and Study design (PICOS) framework: Participants were adults with PTSD or clinically significant PTSD symptoms; Interventions were eligible exercise or movement-based interventions; Comparators were active or inactive control conditions; Outcomes were validated measures of immediate post-treatment PTSD symptom severity; and Study design was restricted to randomized controlled trials.

#### 2.3.1. Participants

Eligible studies enrolled adults with PTSD established either by a formal clinical diagnosis or by clinically significant PTSD symptoms assessed using a validated PTSD-specific instrument. Formal diagnoses based on the Diagnostic and Statistical Manual of Mental Disorders (DSM), the International Classification of Diseases, or a structured clinical interview were considered eligible.

Populations meeting a validated PTSD symptom threshold were also eligible when PTSD represented the principal clinical condition of interest. Studies were excluded when PTSD-specific outcome data could not be distinguished from those of other clinical populations, when participants were exclusively children or adolescents, or when PTSD was not a principal clinical characteristic of the study population.

#### 2.3.2. Exercise and Movement-Based Interventions

Exercise was operationally defined as planned, structured, and repetitive physical activity performed with the purpose of improving or maintaining one or more components of physical fitness or health.

For the strict exercise framework, an intervention was required to include planned and structured physical activity as a primary therapeutic component, with at least one component of frequency, intensity, time, or type sufficiently characterized. Prespecified exercise categories included aerobic exercise, resistance exercise, yoga, and multimodal exercise. Only categories represented by eligible and statistically estimable randomized contrasts were retained as treatment nodes in the fitted network.

The broad exercise and movement-based framework additionally allowed interventions in which bodily movement, stretching, breathing, or another activity-based practice constituted a primary therapeutic component but did not unequivocally satisfy the strict definition of structured physical exercise. Examples included breathing-meditation, mindful stretching, and equine-assisted movement interventions.

Multicomponent mindfulness, psychological, or therapeutic programs were excluded when movement or exercise represented only an ancillary component and its independent therapeutic contribution could not be distinguished from the broader intervention.

The strict and broad frameworks were analyzed separately to examine whether the operational definition of exercise materially affected pooled effects, network geometry, relative treatment estimates, treatment rankings, heterogeneity, or network coherence.

#### 2.3.3. Comparators

Comparator conditions were classified according to their therapeutic content and level of participant engagement.

Waitlist, assessment-only, no-intervention, usual-care, and minimal-contact conditions were classified as inactive controls when they did not provide a structured therapeutic intervention comparable in intensity to the experimental condition.

Conditions providing structured education, attention, social contact, recovery-oriented care, psychological support, or another organized comparator involving meaningful therapeutic or behavioral engagement were classified as active controls. Comparator conditions that included low-dose or ancillary movement or physical activity were also classified as active controls when such activity was not the primary experimental exercise intervention and the condition primarily served as a structured attention, behavioral, or therapeutic comparator. When classification was uncertain, comparator content, contact time, treatment provider, therapeutic intent, and degree of structured participant engagement were considered jointly.

#### 2.3.4. Outcomes

The primary outcome was PTSD symptom severity assessed immediately after completion of the intervention using a validated self-reported or clinician-administered PTSD-specific measure.

When more than one PTSD measure was available within a trial, clinician-administered measures were prioritized when available. Otherwise, a validated self-report measure was selected. When multiple measures of the same assessment type were reported, the outcome designated by the original investigators as the primary PTSD outcome was prioritized.

Outcome directions were harmonized so that higher scores represented greater PTSD symptom severity. Accordingly, negative standardized mean differences (SMDs) indicated greater post-treatment symptom reduction in the exercise or movement-based intervention group than in the comparator group.

Longer-term follow-up outcomes were not included in the primary quantitative analysis because follow-up timing and reporting were insufficiently consistent across studies.

#### 2.3.5. Study Design

Randomized controlled trials were eligible. Individually randomized and cluster-randomized designs were considered eligible when a valid randomized treatment contrast and sufficient quantitative outcome information were available.

Non-randomized studies, uncontrolled pre–post studies, observational studies, case reports, study protocols, and conference abstracts without sufficient quantitative outcome data were excluded. Only reports available in English were eligible for inclusion.

### 2.4. Study Selection

Records identified from the database searches were compiled, and duplicate records were identified by comparing bibliographic information and removed before screening. Titles and abstracts were then screened, followed by full-text assessment of potentially eligible reports.

Study selection was conducted independently by two reviewers. Disagreements were resolved through discussion and consensus between the two reviewers and, when necessary, consultation with an additional reviewer. Reasons for exclusion at the full-text stage were documented and are reported in Supplementary Table S2. The study-selection process was summarized using a PRISMA 2020 flow diagram.

Potentially overlapping reports were evaluated using participant characteristics, recruitment setting, randomized sample size, intervention and comparator groups, intervention duration, outcome measures, and trial-registration information before determining whether reports represented the same randomized cohort.

### 2.5. Data Extraction and Coding Audit

Data were extracted using a standardized study-level extraction form. Extracted information included study and publication characteristics; PTSD eligibility criteria; participant and trauma characteristics; randomized and analyzed sample sizes; age and sex; baseline PTSD severity; intervention type; exercise frequency, intensity, session duration, and program duration; supervision and delivery format; comparator characteristics; concomitant psychological or pharmacological treatment, where reported; PTSD outcome instrument; assessor type; immediate post-treatment means and standard deviations; attrition; adherence; adverse events; and the source and derivation of each effect estimate. Data extraction was conducted independently by two reviewers. Discrepancies were resolved through re-examination of the original reports and consensus between the two reviewers. Study investigators were not contacted for additional or missing data.

When numerical values were reconstructed rather than reported directly, including standard deviations derived from standard errors, confidence intervals, or other statistics, the derivation method and assumptions were documented.

A detailed study-level coding audit was maintained to record randomized and analyzed sample sizes, intervention classification, comparator classification, outcome measure, assessor type, effect-size basis, analytical denominator, eligibility for each quantitative analysis, and verification status.

### 2.6. Effect-Size Calculation and Primary Estimand

Because PTSD symptoms were assessed using different instruments across trials, treatment effects were expressed as standardized mean differences using Hedges’ *g*, with the pooled within-study standard deviation used for standardization and a small-sample correction applied. Negative Hedges’ *g* values indicated lower PTSD symptom severity in the intervention group relative to the comparator [13].

For the primary conventional meta-analysis, eligible randomized comparisons were required to provide sufficient immediate post-treatment arm-level information to estimate a common raw post-treatment effect, including the analyzed sample size, post-treatment mean, and standard deviation for each randomized group. Adjusted model-based estimates, change-score effects, and other alternative estimands were not combined with raw immediate post-treatment arm-level effects in the primary synthesis. When such estimates were the only quantitative results available, they were retained in the alternative-estimand audit but were not combined with the primary quantitative synthesis.

The primary analytical denominator was the number of participants contributing to the selected immediate post-treatment outcome. Randomized and analyzed sample sizes were retained separately throughout the coding process.

### 2.7. Risk-of-Bias Assessment

Risk of bias was assessed using the Cochrane Risk of Bias 2 (RoB 2) tool.

Five domains were evaluated: bias arising from the randomization process; bias due to deviations from intended interventions; bias due to missing outcome data; bias in measurement of the outcome; and bias in selection of the reported result.

Each domain and the overall judgment were classified as low risk, some concerns, or high risk. Particular attention was given to missing post-treatment outcome data, deviations from randomized allocation, self-reported versus clinician-administered PTSD outcomes, and concordance between reported and prespecified analyses.

RoB 2 assessment was conducted independently by two reviewers. Disagreements in domain-level or overall risk-of-bias judgments were resolved through discussion and consensus. Study-level risk-of-bias judgments were subsequently incorporated into the within-study bias domain of CINeMA.

### 2.8. Conventional Pairwise Meta-Analysis

A random-effects meta-analysis was conducted to estimate the average effect of eligible exercise and movement-based interventions on immediate post-treatment PTSD symptom severity relative to comparator conditions. Between-study heterogeneity variance (τ^2^) was estimated using restricted maximum likelihood (REML). The Knapp–Hartung adjustment was applied to the standard error and confidence interval of the pooled effect. Treatment effects were reported as Hedges’ *g* with 95% confidence intervals. Statistical heterogeneity was summarized using Cochran’s (Q), (τ^2^), (I^2^), and (H^2^).

A 95% prediction interval was calculated to estimate the range in which the true effect of a comparable future study might plausibly lie.

The conventional meta-analysis represented the overall exercise and movement-based evidence base. A sensitivity analysis restricted the dataset to interventions satisfying the strict physical-exercise definition.

### 2.9. Subgroup and Sensitivity Analyses

Subgroup and sensitivity analyses were undertaken to examine the robustness of the conventional meta-analysis. Analyses included restriction to interventions meeting the strict physical-exercise definition; subgroup analyses according to comparator intensity (inactive versus active control) and PTSD outcome-assessment method (self-report versus clinician-administered assessment); and a leave-one-study-out sensitivity analysis.

Tests of subgroup differences were interpreted cautiously because several subgroups contained relatively few studies. Lack of statistical significance in a subgroup interaction test was not interpreted as evidence that subgroup effects were identical. Leave-one-study-out analyses were conducted by sequentially excluding each study and re-estimating the random-effects model. An additional sensitivity analysis excluded studies judged to be at high overall risk of bias and repeated both the conventional pairwise meta-analysis and the strict network meta-analysis. For the network analysis, relative-effect estimation, inconsistency assessment, and treatment ranking were repeated using the same analytical framework as in the primary analysis.

### 2.10. Small-Study Effects in the Conventional Meta-Analysis

Potential small-study effects were explored using funnel plots and Egger’s regression test.

Because the number of studies was limited and clinical and methodological heterogeneity was present, funnel-plot asymmetry and Egger-test results were regarded as exploratory and were not interpreted as definitive evidence for or against publication bias.

### 2.11. Network Construction

A frequentist network meta-analysis was conducted using immediate post-treatment PTSD outcomes and effect estimates compatible with the primary raw post-treatment estimand.

The analytical dataset was organized in a contrast-based format, with each record representing an eligible randomized treatment contrast and containing the study identifier, treatment nodes, standardized treatment effect, and corresponding standard error.

The primary network analysis used the strict exercise framework. Prespecified treatment categories comprised aerobic exercise, resistance exercise, yoga, and multimodal exercise, together with active and inactive controls. Only treatment categories represented by eligible and estimable randomized contrasts were retained as nodes in the fitted network.

A broad network sensitivity analysis additionally included eligible movement-based intervention categories that did not unequivocally satisfy the strict exercise definition but represented clinically coherent treatment concepts.

Studies eligible for the systematic review but lacking a compatible randomized contrast or a clinically coherent treatment node were not forced into the network.

Active control was used as the reference treatment for presentation of relative network effects. The reference category affected presentation only and did not alter relative comparisons between treatment pairs.

In network plots, node size was proportional to the number of studies involving each treatment and edge thickness was proportional to the number of randomized direct comparisons between treatment pairs.

### 2.12. Network Meta-Analysis

A frequentist contrast-based random-effects network meta-analysis was fitted under the consistency assumption. A common between-study heterogeneity parameter was assumed across treatment comparisons. No multi-arm trial contributed more than one eligible contrast to either the strict or broad network; therefore, no adjustment for within-study correlation among multiple contrasts was required. Model adequacy was evaluated primarily through assessment of between-study heterogeneity, global inconsistency, local incoherence, and the precision of network estimates rather than through a separate formal goodness-of-fit statistic. Network relative treatment effects were expressed as Hedges’ *g* standardized mean differences with 95% confidence intervals.

Relative effects were evaluated with consideration of effect magnitude, precision, availability of direct and indirect evidence, network geometry, heterogeneity, inconsistency, ranking distributions, and confidence in the evidence. Statistical significance alone was not used to determine comparative treatment superiority.

Because direct head-to-head comparisons between exercise modalities were limited, network estimates relying predominantly on indirect evidence were interpreted cautiously.

### 2.13. Assessment of Transitivity

The transitivity assumption was assessed by examining the distribution of clinically and methodologically relevant potential effect modifiers across direct treatment comparisons.

Potential effect modifiers included, where sufficiently reported, baseline PTSD severity; clinically diagnosed versus symptom-threshold populations; trauma characteristics; participant age and sex; concomitant psychological or pharmacological treatment; intervention frequency, intensity, and duration; supervision; comparator intensity; PTSD assessment instrument; assessor type; analysis population; and handling of missing outcome data.

To improve transparency, the distribution of the principal potential effect modifiers across the observed direct-comparison sets in the strict exercise network was summarized descriptively in Supplementary Table S7. Because several direct comparisons were represented by only one or a small number of trials and reporting of several effect modifiers was incomplete, formal statistical comparisons of their distributions were not considered sufficiently reliable. Transitivity was therefore assessed primarily on the basis of clinical and methodological comparability.

### 2.14. Assessment of Network Inconsistency

Statistical inconsistency was evaluated at both global and local levels.

Global inconsistency was assessed using a random-effects design-by-treatment interaction model.

For treatment comparisons supported by both direct and indirect evidence, local incoherence was evaluated using separating indirect from direct evidence (SIDE) analyses. Direct and indirect estimates, their difference, uncertainty, and corresponding statistical tests were considered jointly. Because the network was sparse, failure to reject a statistical test of inconsistency was not interpreted as evidence of complete network coherence.

### 2.15. Treatment Ranking and Ranking Uncertainty

Treatment-ranking probabilities were estimated from the fitted random-effects consistency model using simulation from the estimated treatment effects and their variance–covariance structure.

A random-number seed of 20,260,816 was specified, and 10,000 simulation draws were used to improve reproducibility.

Because lower PTSD symptom scores represented more favorable outcomes, lower relative treatment effects were assigned better ranks.

For each treatment, the probability of occupying each possible rank, probability of ranking first, mean rank, and surface under the cumulative ranking curve (SUCRA) were calculated.

Treatment rankings were interpreted together with the corresponding relative effect estimates, confidence intervals, amount of direct evidence, network geometry, heterogeneity, inconsistency, and confidence in the evidence. A high probability of ranking first or a high SUCRA value was not interpreted as definitive treatment superiority when the underlying estimates were imprecise, or the comparative evidence was sparse.

### 2.16. Comparison-Adjusted Funnel Plot

Potential small-study effects in the network meta-analysis were explored using a comparison-adjusted funnel plot.

Study-specific standardized mean differences were centered relative to the estimated effect for the corresponding direct comparison after harmonizing treatment-effect direction.

Because the strict network contained a small number of randomized direct comparisons and several treatment pairs were sparsely informed, the comparison-adjusted funnel plot was interpreted as an exploratory assessment rather than as definitive evidence of network-wide publication bias.

### 2.17. Confidence in Network Estimates

Confidence in network relative treatment estimates was evaluated using the Confidence in Network Meta-Analysis (CINeMA) framework.

Six domains were assessed:

1. within-study bias
2. reporting bias
3. indirectness
4. imprecision
5. heterogeneity
6. incoherence

Within-study bias was informed by the RoB 2 assessments and summarized across study contributions using the average risk-of-bias approach. Indirectness was assessed according to the clinical and methodological applicability of studies contributing to each network comparison.

For the incoherence domain, SIDE results were used for comparisons supported by both direct and indirect evidence, whereas the global design-by-treatment interaction test informed judgments for comparisons lacking one of these evidence sources. Following CINeMA guidance, *p* values greater than 0.10 were classified as indicating no concerns regarding incoherence.

An absolute SMD of 0.20 was used as the decision threshold for CINeMA judgments of imprecision and heterogeneity. Because the included trials used multiple PTSD outcome instruments, this value was applied as a common standardized interpretive threshold rather than as a validated PTSD-specific minimal clinically important difference. The same threshold was applied consistently across all network comparisons. Confidence intervals and, where relevant, prediction intervals were evaluated in relation to this threshold when judging imprecision, heterogeneity, and incoherence.

Each CINeMA domain was rated as no concerns, some concerns, or major concerns, and domain-level judgments were integrated to assign an overall confidence rating of high, moderate, low, or very low.

Closely related domains were considered jointly to avoid inappropriate double downgrading for the same underlying source of uncertainty. Treatment rankings and statistical significance were not used to increase confidence in a network estimate.

### 2.18. Broad Exercise and Movement-Based Network Sensitivity Analysis

To assess the sensitivity of comparative-effect and ranking conclusions to the operational definition of exercise, the network meta-analysis was repeated using the broad exercise and movement-based framework.

The broad network incorporated clinically coherent movement-based interventions that did not unequivocally meet the strict exercise definition while retaining the same outcome direction, effect-size framework, reference condition, random-effects structure, ranking procedure, and inconsistency-assessment principles used in the strict network.

Changes in network geometry, relative treatment effects, ranking probabilities, SUCRA values, and inconsistency were examined to determine whether conclusions regarding treatment hierarchy were robust to intervention classification.

### 2.19. Statistical Software

Conventional pairwise and network meta-analyses were conducted using Stata/BE version 19.5 (StataCorp LLC, College Station, TX, USA).

Stata meta-analysis and network meta-analysis procedures were used for random-effects conventional meta-analysis, heterogeneity estimation, forest plots, subgroup analyses, network construction, consistency and inconsistency models, SIDE analyses, comparison-adjusted funnel plots, and treatment-ranking analyses.

CINeMA web application (University of Bern, Bern, Switzerland) was used to evaluate confidence in network relative treatment estimates.

All statistical tests were two-sided. A conventional threshold of (*p* < 0.05) was used for statistical testing; however, heterogeneity, subgroup interaction, inconsistency, and small-study-effect tests were interpreted in conjunction with effect magnitude, precision, network sparsity, and clinical context rather than solely according to statistical significance.

### 2.20. Generative AI Assistance

During manuscript preparation, ChatGPT (GPT-5.6; OpenAI, San Francisco, CA, USA) was used to assist with language refinement, structural organization, statistical coding support, and preparation of graphical materials. All AI-assisted outputs were critically reviewed, verified against the original study data and source materials, and revised as necessary by the authors. No AI-generated output was accepted without author verification, and the authors take full responsibility for the content of the manuscript.

## 3. Results

### 3.1. Study Selection and Characteristics

The study-selection process is summarized in the PRISMA flow diagram (Figure 1). Across the four electronic databases, 37,543 records were identified before deduplication: 494 from PubMed, 6627 from APA PsycINFO, 963 from CENTRAL, and 29,459 from the four ScienceDirect searches combined. Manual backward citation searching did not identify any additional eligible studies.

Detailed characteristics of the included randomized trials are presented in Table 1 [27,28,29,30,31,32,33,34,35,36,37,38,39,40,41,42,43,44,45,46], and study-level extraction and coding decisions are provided in Supplementary Table S3. The included studies represented clinically heterogeneous adult populations, including military veterans and active-duty personnel, civilians and community samples, psychiatric inpatients, former combatants, trauma-affected refugees, nurses, and women with interpersonal- or stillbirth-related trauma. PTSD eligibility ranged from formally diagnosed DSM-based PTSD and chronic or treatment-resistant PTSD to partial/subthreshold, probable, or validated symptom-threshold PTSD. Participant age also varied substantially across studies. For example, the Seppälä et al. trial included relatively young veterans (mean age 28.1 years in the intervention group and 29.2 years in the control group), whereas mean ages were 44.4 years in Mitchell et al., 47.8 years in Rosenbaum et al., and approximately 58 years in Carter et al. Because age and baseline symptom severity were reported using heterogeneous summary formats and PTSD instruments, these characteristics were not pooled across studies. Baseline symptom burden nevertheless reflected clinically meaningful PTSD in many samples; for example, mean baseline Clinician-Administered PTSD Scale (CAPS) scores were approximately 56 in Carter et al., whereas mean baseline PTSD Checklist–Civilian Version (PCL-C) scores were approximately 64 in Rosenbaum et al. Other studies enrolled participants using validated symptom thresholds, including the Clinician-Administered PTSD Scale for DSM-5 (CAPS-5) or PTSD Checklist for DSM-5 (PCL-5) criteria.

Intervention content and dose were likewise heterogeneous. The strict exercise interventions included yoga, resistance exercise, aerobic exercise, and multimodal exercise programs, while the broad exercise and movement-based framework additionally included breathing–meditation and mindful stretching. Intervention duration ranged from short intensive or brief programs to longer structured interventions: examples included a 7-day intensive breathing-based meditation program, a 3-week high-intensity resistance-training program, yoga programs lasting approximately 6–16 weeks, and multimodal programs generally lasting about 10–12 weeks; the pairwise evidence base also included a 12-month nature-adventure rehabilitation program. Within the strict exercise network, interventions lasted 3–16 weeks and, where reported, were generally delivered one to three times per week for approximately 30–90 min per session, although some programs incorporated home practice, walking, or self-directed activity. Comparator conditions also varied in therapeutic intensity, and both self-reported and clinician-administered PTSD outcomes were represented.

Of the 20 studies included in the systematic review, 15 contributed to the primary pairwise meta-analysis. Four otherwise eligible randomized trials (Powers et al., Huberty et al., Young-McCaughan et al., and Bryant et al.) reported only change-based or adjusted/model-based estimates that could not be converted to the prespecified raw immediate post-treatment Hedges’ *g* without additional assumptions and were therefore not pooled with the primary quantitative synthesis. A structured audit of these four trials is presented in Supplementary Table S8; their findings were directionally mixed and did not indicate a consistent pattern that would materially alter the interpretation of the primary raw-post synthesis. Johnson et al. used a randomized waitlist crossover design, but the reported post-riding statistics pooled participants across treatment periods; consequently, a clean independent randomized-period contrast could not be reconstructed.

Of the 15 studies contributing to the primary pairwise meta-analysis, 11 also contributed to the strict exercise network. Carter et al., Kim et al., and Seppälä et al. were eligible under the broad exercise and movement-based framework but did not satisfy the prespecified strict physical-exercise definition, whereas the nature-adventure rehabilitation intervention evaluated by Gelkopf et al. contributed to the pairwise analysis but could not be mapped to a prespecified clinically coherent network node. Consequently, 11 randomized direct comparisons contributed to the strict exercise network, whereas 14 contributed to the broad exercise and movement-based network sensitivity analysis.

Risk-of-bias assessment indicated that 3 of the 20 included studies were judged to be at low overall risk of bias, 12 had some concerns, and 5 were judged to be at high risk of bias (Supplementary Table S4; Supplementary Figures S1 and S2). High-risk judgments were driven primarily by concerns regarding missing outcome data, while concerns regarding deviations from intended interventions, outcome measurement, and selective reporting were also common.

### 3.2. Conventional Pairwise Meta-Analysis

The primary conventional random-effects meta-analysis included 15 randomized trials, with 863 participants contributing to the selected immediate post-treatment analyses. Exercise and movement-based interventions were associated with a statistically significant reduction in PTSD symptom severity compared with control conditions (Hedges’ *g* = −0.49, 95% confidence interval (CI) −0.69 to −0.28; Figure 2).

Between-study heterogeneity was moderate (τ^2^ = 0.050; I^2^ = 40.2%; H^2^ = 1.67). Cochran’s test of heterogeneity did not reach the conventional threshold for statistical significance (Q(14) = 23.20, *p* = 0.057). Using REML estimation with the Knapp–Hartung adjustment, the overall treatment effect was statistically significant (t(14) = −5.13, *p* < 0.001).

The 95% prediction interval ranged from −1.01 to 0.04, crossing the null value. Thus, although the average treatment effect favored exercise and movement-based interventions, the prediction interval indicated that a negligible or null effect remained plausible in a future clinically comparable trial.

### 3.3. Subgroup and Sensitivity Analyses

Restriction to interventions satisfying the strict physical-exercise definition produced an effect estimate similar to that of the overall analysis. Across 11 trials meeting the strict physical-exercise definition, the pooled effect was approximately *g* = −0.44 (95% CI −0.66 to −0.20). The four interventions not meeting the strict definition produced a larger point estimate (*g* = −0.68, 95% CI −1.32 to −0.04); however, the between-group difference was not statistically significant (Q(1) = 1.12, *p* = 0.29).

Comparator intensity showed a similar pattern. Exercise and movement-based interventions compared with inactive controls produced a larger pooled effect (k = 11; *g* = −0.59, 95% CI −0.82 to −0.36) than those compared with active controls (k = 4; *g* = −0.27, 95% CI −0.81 to 0.27). The test of subgroup differences was not statistically significant (Q(1) = 2.44, *p* = 0.12).

Effects also differed numerically according to outcome-assessment method. Studies using self-reported PTSD outcomes showed a pooled effect of approximately *g* = −0.60 (95% CI −0.88 to −0.32; k = 9), whereas clinician-administered outcomes showed a smaller effect (*g* = −0.33, 95% CI −0.66 to 0.01; k = 6). The between-group difference was not statistically significant (Q(1) = 2.10, *p* = 0.15).

Leave-one-study-out analyses indicated that the overall finding was not driven by any single trial. Sequential omission of individual studies produced pooled estimates ranging approximately from *g* = −0.56 to −0.45, and all resulting confidence intervals remained on the side favoring exercise. These findings supported the robustness of the direction and magnitude of the primary pairwise result.

### 3.4. Geometry of the Strict Exercise Network

The strict network comprised 11 randomized direct comparisons across five treatment nodes, with 754 participants contributing to the network analysis: active control, inactive control, multimodal exercise, resistance exercise, and yoga (Figure 3).

Five unique direct treatment-pair comparisons were represented. Yoga was directly compared with active control in two studies and with inactive control in four studies. Multimodal exercise was compared with active control in one study and with inactive control in three studies, whereas resistance exercise was connected to active control through a single direct comparison.

The network contained a closed loop involving active control, inactive control, yoga, and multimodal exercise. Importantly, no direct head-to-head comparisons were available between the exercise modalities themselves. Consequently, comparisons among exercise modalities depended predominantly on indirect evidence through the control nodes.

Potential effect modifiers showed appreciable clinical and methodological variation across the direct-comparison sets (Supplementary Table S7). Differences were observed, where reported, in PTSD eligibility and diagnostic status, participant and trauma characteristics, intervention exposure, comparator content and concomitant care, and outcome-assessment methods. Because several direct-comparison sets were represented by only one or a small number of trials and reporting of key effect modifiers was incomplete, formal statistical comparison of their distributions was not considered reliable. Transitivity was therefore considered plausible but uncertain rather than firmly established.

### 3.5. Network Relative Treatment Effects

Relative effects from the random-effects consistency model are presented in Table 2. Relative to active control, yoga was associated with an estimated standardized mean difference of −0.32 (95% CI −0.73 to 0.08), multimodal exercise with −0.13 (95% CI −0.63 to 0.37), and resistance exercise with −0.32 (95% CI −1.32 to 0.68).

None of these estimates provided statistically clear evidence that an individual exercise modality was superior to active control because all corresponding 95% confidence intervals included the null value. The estimate for resistance exercise was particularly imprecise, reflecting the limited evidence contributing to that treatment node.

Likewise, the available network evidence did not demonstrate statistically clear superiority of one exercise modality over another. Because direct head-to-head trials between exercise modalities were absent, these comparisons were largely dependent on indirect evidence and were interpreted cautiously. Accordingly, the network estimates should be regarded as exploratory comparative evidence rather than as firm evidence of relative superiority among exercise modalities.

### 3.6. Network Inconsistency

In the strict network, the global design-by-treatment interaction test did not indicate statistically significant evidence of inconsistency (χ^2^(1) = 2.35, *p* = 0.125). SIDE analyses were evaluable for four comparisons supported by both direct and indirect evidence: active control versus multimodal exercise, active control versus yoga, inactive control versus multimodal exercise, and inactive control versus yoga. None showed statistically significant direct–indirect disagreement (all *p* = 0.125). The active control versus resistance exercise comparison could not be evaluated using SIDE because resistance exercise was connected to the network through a single direct comparison and lacked an independent indirect pathway. Given the sparsity of the network and the limited number of studies informing several comparisons, the absence of statistically significant inconsistency was not interpreted as evidence of complete network coherence.

### 3.7. Treatment Ranking in the Strict Network

Treatment-ranking results are presented in Table 3 and Figure 4. Resistance exercise had the highest probability of ranking first (47.9%), followed by yoga (40.4%) and multimodal exercise (10.6%). Active and inactive controls had probabilities of ranking first of 1.1% and 0.0%, respectively.

However, ranking-first probability and overall rank distribution did not identify the same treatment as most favorable. When the full ranking distribution was considered, yoga had the most favorable mean rank (1.8) and the highest SUCRA value (0.809). Resistance exercise had a mean rank of 2.3 and a SUCRA value of 0.685, whereas multimodal exercise had a mean rank of 2.7 and a SUCRA value of 0.563. Active and inactive controls had mean ranks of 3.5 and 4.8 and SUCRA values of 0.383 and 0.059, respectively.

The rankograms demonstrated substantial uncertainty in the treatment hierarchy. Although resistance exercise had the greatest probability of occupying rank 1, its ranking distribution was more dispersed, whereas yoga was concentrated more consistently among the favorable ranks. Therefore, probability of ranking first did not directly correspond to the most consistently favorable overall ranking.

### 3.8. Confidence in the Network Evidence

Confidence in relative treatment effects was evaluated using CINeMA. Across the 10 possible pairwise comparisons within the five-node strict network, overall confidence was rated very low.

The main concerns involved imprecision, within-study bias, and reporting bias, with additional concerns regarding heterogeneity for selected comparisons. Incoherence was rated as no concerns across the network under the CINeMA framework, consistent with the absence of statistically significant global or local inconsistency signals.

The very low confidence ratings indicate substantial uncertainty surrounding the comparative treatment estimates. Accordingly, favorable SUCRA values and high probabilities of ranking first should be regarded as hypothesis-generating summaries of a highly uncertain comparative evidence base rather than as evidence of definitive superiority, and should not be used to recommend one exercise modality over another.

### 3.9. Sensitivity Analysis Excluding Studies at High Risk of Bias

After excluding four studies judged to be at high overall risk of bias, 11 trials involving 616 participants remained in the conventional meta-analysis. The pooled effect remained similar to the primary estimate (Hedges’ *g* = −0.47, 95% CI −0.73 to −0.21; τ^2^ = 0.066), although the 95% prediction interval continued to cross the null (−1.11 to 0.17).

In the corresponding strict network sensitivity analysis, the strict network was reduced from 11 to 8 randomized direct comparisons, comprising 549 participants while retaining all five treatment nodes. No exercise modality demonstrated statistically clear superiority over active control (multimodal exercise: *g* = −0.11, 95% CI −0.73 to 0.52; resistance exercise: *g* = −0.32, 95% CI −1.40 to 0.76; yoga: *g* = −0.22, 95% CI −0.85 to 0.41). The global design-by-treatment interaction remained non-significant (χ^2^(1) = 2.45, *p* = 0.118), and none of the four evaluable SIDE comparisons showed statistically significant direct–indirect disagreement (all *p* = 0.118). Resistance exercise retained the highest probability of ranking first (51.0%), whereas resistance exercise and yoga had identical mean ranks (2.2) and similar SUCRA values (approximately 0.7). Thus, exclusion of high-risk-of-bias studies did not materially alter the principal comparative conclusion that the available evidence does not support a stable treatment hierarchy.

### 3.10. Broad Exercise and Movement-Based Network Sensitivity Analysis

The broad exercise and movement-based network expanded the intervention definition to include additional clinically coherent movement-based approaches. The network comprised 14 randomized direct comparisons involving 821 participants across seven treatment nodes: active control, inactive control, multimodal exercise, resistance exercise, yoga, breathing–meditation, and mindful stretching.

Relative to active control, the estimated effects were −0.34 (95% CI −1.15 to 0.48) for breathing–meditation, −0.13 (95% CI −0.61 to 0.35) for multimodal exercise, −1.05 (95% CI −2.17 to 0.07) for mindful stretching, −0.32 (95% CI −1.30 to 0.66) for resistance exercise, and −0.32 (95% CI −0.71 to 0.07) for yoga. None of these estimates reached the conventional threshold for statistical significance.

Treatment ranking changed substantially when the broader intervention definition was applied. Mindful stretching had a 75.7% probability of ranking first, a mean rank of 1.5, and a SUCRA value of 0.922. Yoga had a probability of ranking first of 2.8% and SUCRA of 0.638; breathing–meditation, 7.7% and 0.604; resistance exercise, 13.3% and 0.566; and multimodal exercise, 0.5% and 0.433.

Despite the highly favorable ranking of mindful stretching, its relative effect estimate was imprecise and its confidence interval crossed the null. The substantial change in ranking between the strict and broad networks therefore demonstrated that treatment rankings were sensitive to the operational definition and classification of interventions.

In the currently reported broad network sensitivity analysis, the global design-by-treatment inconsistency test was not statistically significant (χ^2^(1) = 2.57, *p* = 0.109). SIDE analyses for active control versus multimodal exercise, active control versus yoga, inactive control versus multimodal exercise, and inactive control versus yoga likewise showed no statistically significant direct–indirect disagreement (all *p* = 0.109).

These findings indicated that expanding the network altered treatment-ranking distributions considerably, even though the available broad network analysis did not provide statistically clear evidence of global or local inconsistency.

### 3.11. Small-Study Effects and Reporting Bias

A comparison-adjusted funnel plot was constructed for the strict exercise network (Supplementary Figure S6). Because the network contained only 11 randomized direct comparisons and several treatment pairs were informed by very few studies, the funnel plot was regarded as exploratory rather than as a definitive assessment of small-study effects or publication bias.

For the conventional pairwise meta-analysis, visual inspection of the funnel plot did not indicate marked asymmetry, and Egger’s regression test provided no statistically significant evidence of small-study effects (*β* = −0.12, SE = 1.03, z = −0.11, *p* = 0.910; Supplementary Figure S9). Given the limited number of included trials, these findings were interpreted cautiously and were not considered evidence that reporting bias was absent.

## 4. Discussion

### 4.1. Principal Findings

This systematic review and network meta-analysis addressed two related but distinct questions: whether exercise and movement-based interventions improve post-traumatic stress disorder (PTSD) symptoms on average, and whether the available comparative evidence is sufficiently robust to support a clinically meaningful hierarchy of specific exercise modalities. The conventional random-effects meta-analysis of 15 randomized trials involving 863 participants showed a moderate reduction in PTSD symptom severity in favor of exercise and movement-based interventions (Hedges’ *g* = −0.49, 95% CI −0.69 to −0.28). This finding was reasonably robust to analytical restriction. When the analysis was limited to interventions satisfying the strict physical-exercise definition, the pooled effect remained similar in direction and magnitude (*g* ≈ −0.44, 95% CI −0.66 to −0.20). Leave-one-study-out analyses likewise produced consistently favorable estimates, indicating that the overall finding was not dependent on a single influential trial.

Nevertheless, the 95% prediction interval for the primary meta-analysis ranged from −1.01 to 0.04 and therefore crossed the null. This distinction between the average pooled effect and the range of effects expected across future settings is clinically important [26]. Although the available evidence supports an average reduction in PTSD symptoms, it does not imply that a clinically meaningful benefit will necessarily be observed in every population, intervention setting, or future trial.

The network meta-analysis provided substantially less support for comparative superiority among specific exercise modalities. The strict exercise network comprised 11 randomized direct comparisons involving 754 participants across five treatment nodes: active control, inactive control, multimodal exercise, resistance exercise, and yoga. Importantly, no direct head-to-head comparisons were available between the exercise modalities themselves. Consequently, comparisons among exercise modalities depended largely on indirect evidence through control conditions.

Relative to active control, the estimated effects for yoga, multimodal exercise, and resistance exercise all favored exercise numerically, but all corresponding 95% confidence intervals included the null. Thus, the available network evidence did not demonstrate statistically clear superiority of any individual exercise modality.

Treatment-ranking statistics further illustrated the uncertainty of the comparative evidence. Resistance exercise had the highest probability of ranking first (47.9%), whereas yoga had the most favorable mean rank (1.8) and the highest SUCRA value (0.809). The fact that different ranking metrics identified different apparently favorable treatments is itself informative. Rather than supporting a stable treatment hierarchy, the rankings indicate substantial uncertainty about the relative position of individual exercise modalities [15,47].

This interpretation was reinforced by the CINeMA assessment, in which confidence was rated very low for all 10 pairwise comparisons in the strict five-node network. Accordingly, the principal finding of this study is not that a particular form of exercise should be considered superior for PTSD. Rather, exercise and movement-based interventions appear beneficial on average, while the current comparative evidence remains insufficient to establish a reliable hierarchy among specific exercise modalities.

### 4.2. Exercise in Clinical Decision-Making for PTSD

The distinction between average treatment benefit and comparative superiority is central to the clinical interpretation of the present findings. The conventional meta-analysis supports a potential average benefit of exercise and movement-based interventions, whereas the network meta-analysis did not establish the superiority of any specific exercise modality. Accordingly, appropriately structured exercise may be considered as an adjunctive component of PTSD care rather than as a replacement for established trauma-focused psychological or indicated pharmacological treatment [11,48]. Because the present analysis did not directly compare exercise with established first-line PTSD treatments, equivalence or superiority cannot be inferred. Modality-specific decisions should therefore be individualized according to clinical context and patient characteristics [48,49].

### 4.3. Patient-Centered Selection of Exercise Interventions

Given the absence of a reliable population-level treatment hierarchy, modality selection should emphasize patient-level considerations rather than ranking position. PTSD populations differ in trauma history, symptom severity, physical health, comorbidity, previous exercise exposure, and treatment preference [4,50], all of which may influence feasibility and sustained participation [51,52]. Treatment rankings summarize relative positions within an analyzed network and should not be interpreted as identifying the optimal intervention for an individual patient [15]. Accordingly, exercise selection should consider clinical suitability, safety, feasibility, accessibility, sustainability, and patient preference [4,50,53]. Future comparative trials should evaluate adherence, acceptability, and treatment preference alongside PTSD symptom outcomes.

### 4.4. Why Probabilistic Rankings Require Cautious Interpretation

Treatment-ranking statistics are intuitively appealing because they summarize a complex network of comparisons into an apparently simple hierarchy. However, rankings can become unstable when evidence networks are sparse, direct comparisons are limited, and relative-effect estimates are imprecise [15,47]. All of these conditions were present in the current strict network.

Resistance exercise had the highest probability of ranking first at 47.9%, whereas yoga had a lower probability of ranking first at 40.4% but the most favorable mean rank and highest SUCRA value. Resistance exercise also had a particularly wide confidence interval for its relative effect versus active control. Thus, a high probability of occupying rank 1 did not correspond to the most consistently favorable position across the full ranking distribution.

This divergence illustrates an important limitation of ranking statistics. Probability of being best emphasizes the chance of occupying a single extreme position, whereas SUCRA and mean rank summarize the distribution across multiple possible ranks [15,54]. When relative-effect estimates are imprecise, apparently favorable rankings may therefore coexist with substantial uncertainty regarding true comparative superiority [47].

The broad sensitivity analysis reinforced this concern because the identity of the highest-ranked intervention changed when the network definition was expanded. This classification sensitivity is examined further in the context of intervention definition and network robustness in Section 4.5.

Taken together, these findings show that an apparently favorable ranking can coexist with substantial statistical uncertainty. Rankings should therefore be interpreted alongside relative-effect estimates, confidence intervals, network geometry, the amount of direct evidence, risk of bias, and confidence in the evidence. Reporting or applying treatment rankings in isolation may convey a degree of certainty that the underlying evidence does not support [15,24,55].

### 4.5. Intervention Definition, Transitivity, and Network Robustness

One of the most informative findings of the present study was the sensitivity of the apparent treatment hierarchy to the operational definition of exercise. The strict analysis was restricted to interventions that more clearly satisfied the definition of structured physical exercise, whereas the broad sensitivity analysis additionally incorporated clinically coherent movement-based approaches such as breathing–meditation and mindful stretching [15,56].

Broadening from the strict exercise network to the broad exercise and movement-based network changed the network geometry from 11 direct comparisons across five nodes (754 participants) to 14 direct comparisons across seven nodes (821 participants) and, importantly, changed the identity of the highest-ranked intervention. Because this shift occurred despite considerable imprecision in the relative-effect estimates, it indicates sensitivity not only to sampling variation but also to analytical decisions concerning intervention classification. Increasing network size or connectivity does not necessarily improve the clinical credibility of a treatment hierarchy when additional nodes differ in therapeutic components, physical demands, or intended mechanisms [15,56]. Accordingly, the sensitivity of the treatment hierarchy to node definition should be considered a fundamental limitation of the comparative conclusions rather than merely a secondary sensitivity-analysis finding. The structured assessment of potential effect modifiers (Supplementary Table S7) further showed clinical and methodological variation across direct-comparison sets in PTSD eligibility and diagnostic status, population and trauma characteristics, intervention exposure, comparator content and concomitant care, and outcome-assessment methods. These differences do not by themselves demonstrate a violation of transitivity; however, together with sparse direct evidence and incomplete reporting, they limit confidence that the transitivity assumption is fully satisfied.

The broad network global inconsistency test did not provide statistically significant evidence of inconsistency (χ^2^(1) = 2.57, *p* = 0.109), and the evaluable SIDE comparisons similarly did not demonstrate statistically clear direct–indirect disagreement. However, statistical coherence alone does not establish that the transitivity assumption is clinically secure. In a sparse network, inconsistency tests may have limited ability to identify meaningful violations [15,57,58], and the substantial change in treatment ranking after modifying the intervention definition remains relevant to interpretation.

In the strict network, neither the global design-by-treatment interaction test (χ^2^(1) = 2.35, *p* = 0.125) nor the four evaluable SIDE analyses (all *p* = 0.125) provided statistically significant evidence of inconsistency. However, statistical non-significance should not be interpreted as confirmation of network coherence. The strict network was sparse, no direct head-to-head comparisons were available between exercise modalities, and several treatment contrasts were informed by only one or a small number of trials. Consequently, uncertainty regarding the transitivity and coherence assumptions remains relevant when interpreting relative effects and treatment rankings [57,58].

These findings emphasize that intervention classification in network meta-analysis should be determined primarily by clinically defensible definitions rather than by the analytical convenience of increasing network connectivity [15,56].

### 4.6. Implications for Healthcare Delivery and Guideline Development

Guideline panels and healthcare providers should distinguish between recommending structured exercise as an adjunctive intervention and claiming superiority of a specific exercise modality; the current evidence supports the former more clearly than the latter. Ranking metrics do not capture important implementation considerations such as feasibility, acceptability, patient preference, and clinical context [4,47,59]. Until stronger direct comparative evidence becomes available, healthcare systems may therefore support access to safe and appropriately structured exercise options while allowing individualized modality selection.

### 4.7. Implications for Future Comparative-Effectiveness Research

The principal limitation of the current comparative evidence base is not simply the total number of randomized trials but the absence of direct head-to-head comparisons between exercise modalities. Most trials compared an exercise intervention with an active or inactive control, leaving the relative effectiveness of different forms of exercise dependent largely on indirect evidence.

Future randomized trials should therefore prioritize direct comparisons between clinically relevant exercise modalities. Within the treatment categories represented in the current strict network, particularly informative comparisons would include yoga versus resistance exercise, yoga versus multimodal exercise, and resistance versus multimodal exercise. Additional well-powered trials of clearly defined aerobic exercise would also be valuable for establishing its position within future comparative networks.

Interventions should be described in sufficient detail to permit clinically meaningful classification, replication, and implementation, including frequency, intensity, session duration, program duration, progression, supervision, delivery format, and adherence, in accordance with established intervention- and exercise-reporting guidance [60,61]. Comparator conditions should likewise be described in sufficient detail to distinguish inactive controls from structured attention, educational, psychological, or other active therapeutic comparators [61].

Future studies should standardize PTSD outcome assessment and prospectively specify primary outcomes and assessment time points to facilitate comparison and evidence synthesis across trials [62]. Longer-term follow-up is particularly important because the present analysis focused primarily on immediate post-treatment symptom severity and therefore cannot establish the durability of exercise-related improvements.

Adherence, dropout, adverse events, acceptability, quality of life, physical-health outcomes, and concomitant psychological or pharmacological treatment should also be reported systematically; in particular, harms should be prespecified, assessed, and reported transparently [63]. Adequately powered investigations of potential effect modifiers—including baseline PTSD severity, trauma characteristics, sex, age, physical comorbidity, and medication use—may ultimately provide more clinically useful information for individualized exercise prescription and would also strengthen assessment of transitivity in future comparative networks [15,64].

### 4.8. Strengths and Limitations

This study has several strengths. First, it explicitly distinguished the overall effect of exercise from the more demanding question of comparative superiority among individual exercise modalities. Second, the primary quantitative synthesis used a common immediate post-treatment raw-data estimand rather than combining fundamentally different effect estimands within the same primary model. Third, robustness was evaluated through restriction to the strict physical-exercise definition, subgroup and leave-one-study-out analyses, and a broad exercise and movement-based network sensitivity analysis. Fourth, network interpretation incorporated direct and indirect evidence, network geometry, ranking distributions, and confidence in the evidence rather than relying on statistical significance or a single ranking statistic.

Several limitations should also be acknowledged. First, the strict network was sparse and contained no direct head-to-head comparisons between exercise modalities. Several treatment contrasts were informed by only one or a small number of trials, resulting in substantial imprecision and dependence on indirect evidence.

Second, the structured assessment of potential effect modifiers across direct-comparison sets (Supplementary Table S7) identified clinical and methodological variation in PTSD eligibility and diagnostic status, participant and trauma characteristics, intervention exposure, comparator content and concomitant care, and outcome-assessment methods. Because several comparison sets were informed by only one or a few trials and reporting of key modifiers was incomplete, transitivity could not be demonstrated empirically and was considered plausible but uncertain. This uncertainty limits confidence in the indirect relative-treatment estimates and the treatment rankings derived from them.

Third, although restricting the primary quantitative synthesis to a common raw immediate post-treatment estimand improved comparability across studies, it necessarily reduced the randomized evidence contributing to the pooled and network analyses. Four otherwise eligible randomized trials reported only change-based or adjusted/model-based estimates that could not be converted to the prespecified raw-post Hedges’ *g* without additional assumptions (Supplementary Table S8). The alternative-estimand audit showed no consistent pattern that would materially alter the interpretation of the primary raw-post synthesis. Powers et al. reported a favorable exercise-augmentation effect, Bryant et al. showed a numerically favorable immediate post-treatment estimate and a favorable primary follow-up effect, whereas Young-McCaughan et al. found no significant between-condition differences and Huberty et al. reported favorable numerical trends without statistically significant comparative PTSD effects. Accordingly, these trials were retained in a structured alternative-estimand audit rather than pooled with the primary quantitative synthesis. Nevertheless, selection according to the availability and reporting format of outcome data cannot be excluded as a potential source of bias.

Fourth, subgroup analyses according to comparator intensity and outcome-assessment method were based on relatively few studies. Although effects were numerically larger against inactive controls than active controls and for self-reported than clinician-administered outcomes, the corresponding between-group differences were not statistically significant. These findings should therefore be regarded as exploratory, and the absence of statistically significant interaction should not be interpreted as evidence of equivalence between subgroups.

Fifth, the small evidence base limited the reliability of assessments of small-study effects and reporting bias. Funnel-plot and related analyses should therefore be considered exploratory.

Sixth, the review was restricted to English-language reports, which may have resulted in the omission of otherwise eligible evidence published in other languages.

Seventh, the analysis focused primarily on PTSD symptom severity immediately after treatment. It did not establish longer-term effectiveness, comparative safety, adherence, acceptability, cost-effectiveness, or effects on broader patient-centered outcomes.

Finally, the overall risk-of-bias profile limits confidence in both the pooled pairwise effect and the comparative network estimates. Only three of the 20 included studies were judged to be at low overall risk of bias, whereas 12 had some concerns and five were judged to be at high risk, with missing outcome data representing the principal source of high-risk judgments. Although exclusion of high-risk studies yielded a similar pooled pairwise effect (*g* = −0.47, 95% CI −0.73 to −0.21) and did not materially alter the conclusions of the strict network analysis, these sensitivity findings do not eliminate the possibility that bias in the underlying trials affected the magnitude or precision of the estimated effects. Within-study bias also contributed to the CINeMA assessments, and confidence in all network comparisons remained very low.

## 5. Conclusions

Exercise and movement-based interventions were associated with a moderate average reduction in PTSD symptom severity, and the direction and magnitude of the pooled effect remained similar when the analysis was restricted to interventions meeting a strict physical-exercise definition. However, the 95% prediction interval crossed the null, indicating that treatment effects may vary considerably across clinical populations and settings. Because the analysis was restricted to immediate post-treatment PTSD severity, these findings do not establish whether symptom improvements are sustained over time.

The evidence was substantially less certain regarding the comparative effectiveness of individual exercise modalities. No exercise modality demonstrated robust superiority in the strict exercise network, no direct head-to-head comparisons were available between exercise modalities, transitivity could not be firmly established, and confidence in all network comparisons was rated very low.

Moreover, resistance exercise had the highest probability of ranking first, whereas yoga had the most favorable SUCRA value and mean rank, and the apparent treatment hierarchy changed when the broad exercise and movement-based framework was applied.

These findings indicate that probabilistic treatment rankings should not be interpreted as a stable clinical hierarchy. In particular, SUCRA, mean rank, and probability-of-being-best estimates should not be used in isolation to determine which exercise modality should be recommended for PTSD.

At present, the available evidence supports consideration of safe and appropriately structured exercise as a potential adjunctive component of patient-centered PTSD care, while modality selection should reflect clinical suitability, safety, feasibility, accessibility, sustainability, and patient preference. Stronger conclusions regarding the comparative superiority of specific exercise modalities will require adequately powered head-to-head randomized trials with standardized intervention definitions, comparator conditions, outcome assessments, and longer-term patient-centered follow-up.

## Figures and Tables

**Figure 1:**
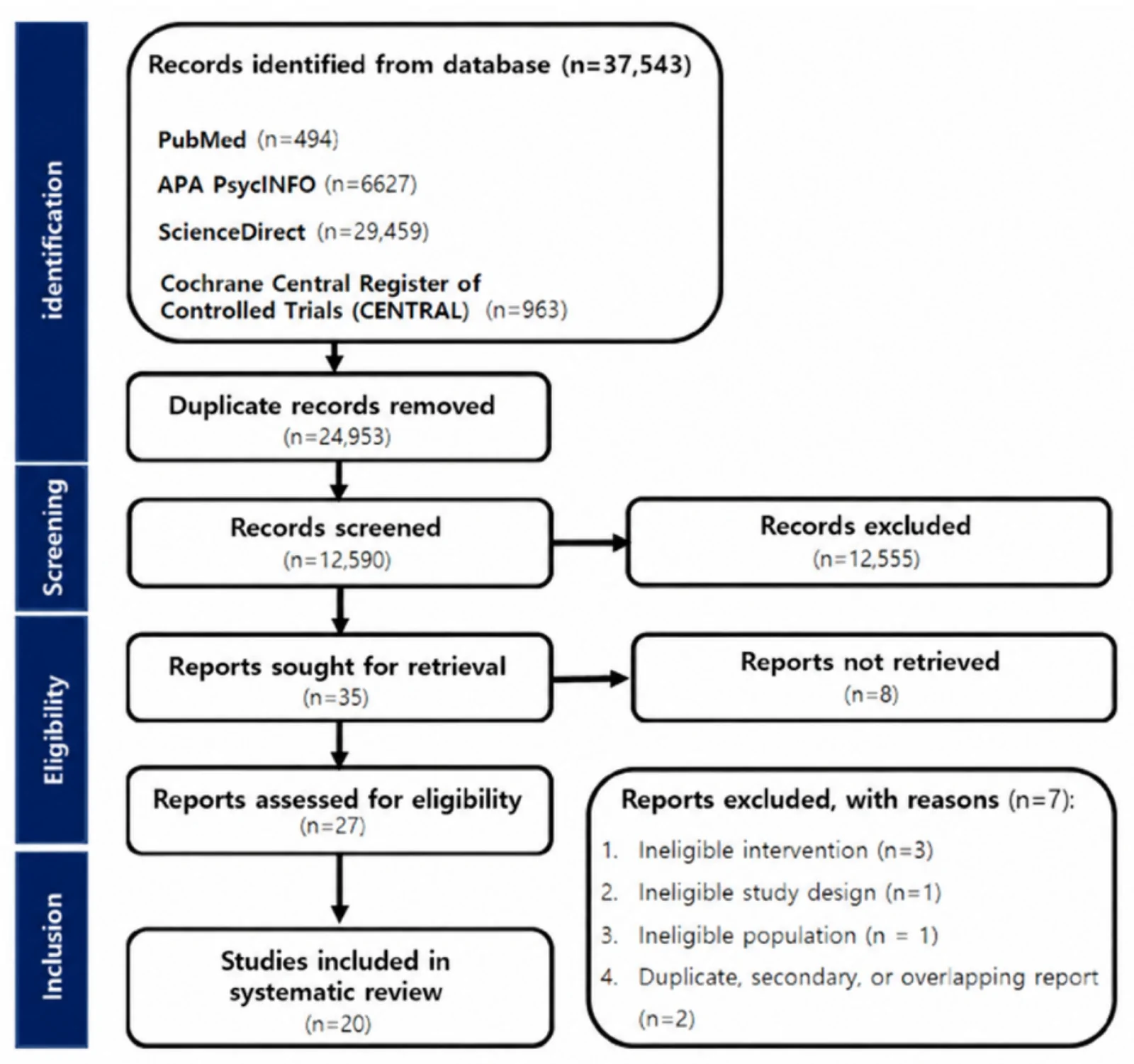
Preferred Reporting Items for Systematic Reviews and Meta-Analyses (PRISMA) 2020 flow diagram of study selection. Twenty studies were included in the systematic review; 15 contributed to the primary pairwise meta-analysis, 11 to the strict exercise network meta-analysis, and 14 to the broad exercise and movement-based network sensitivity analysis.

**Figure 2:**
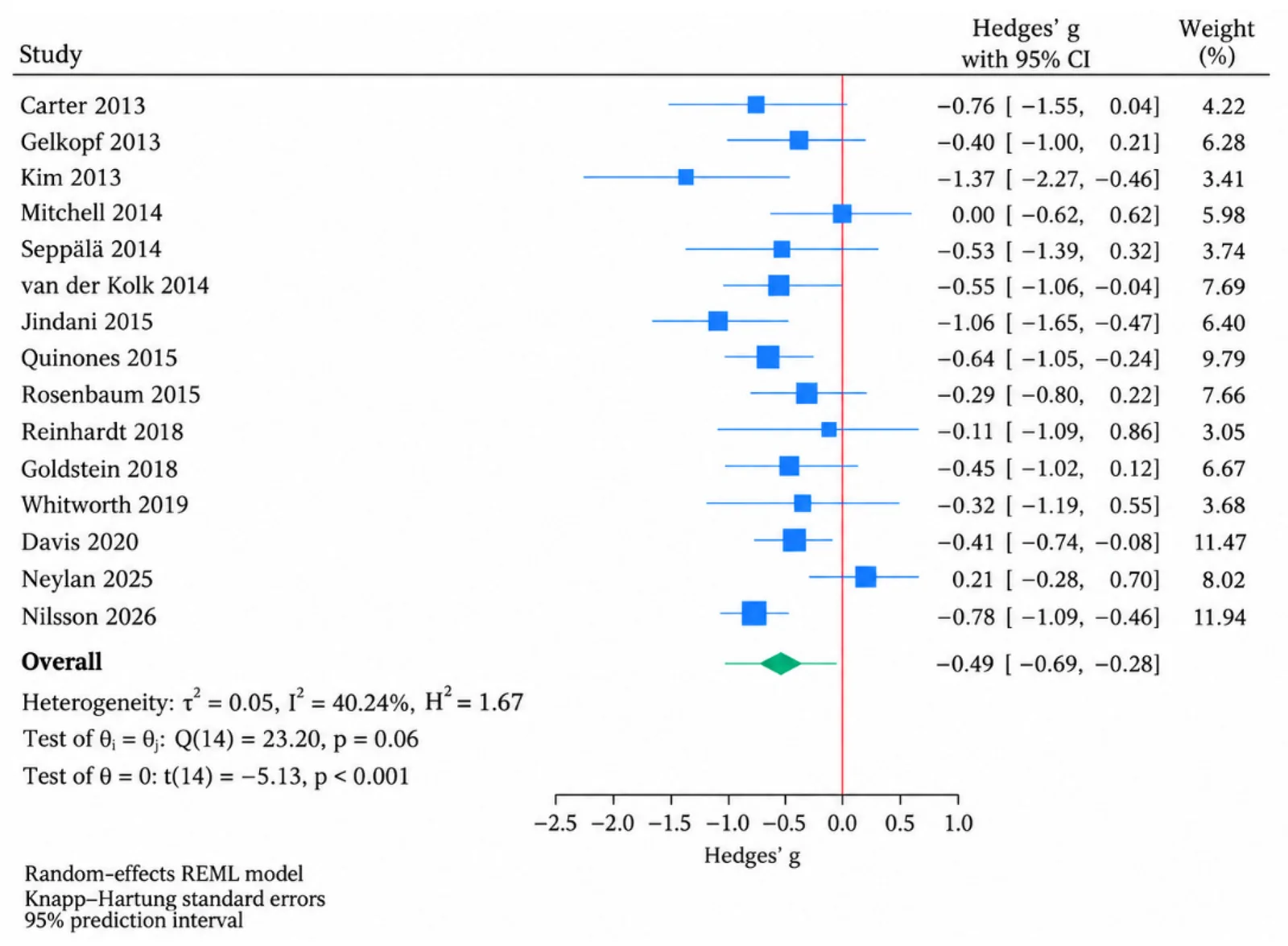
Forest plot of the overall effect of exercise and movement-based interventions on PTSD symptoms. Study-specific effects for the 15 contributing trials [27,29,30,31,32,33,36,37,38,40,41,43,44,45,46] and pooled effects are presented as Hedges’ *g* with 95% confidence intervals. The pooled effect was estimated using a random-effects model with restricted maximum likelihood (REML) estimation and Knapp–Hartung adjustment. Negative values indicate greater reductions in PTSD symptoms in the intervention group. The horizontal line extending beyond the pooled-effect diamond represents the 95% prediction interval. Blue squares and horizontal lines indicate study-specific Hedges’ *g* estimates and 95% confidence intervals, with square size reflecting study weight. The green diamond represents the pooled effect, the green horizontal line represents the 95% prediction interval, and the red vertical line denotes the null value.

**Figure 3:**
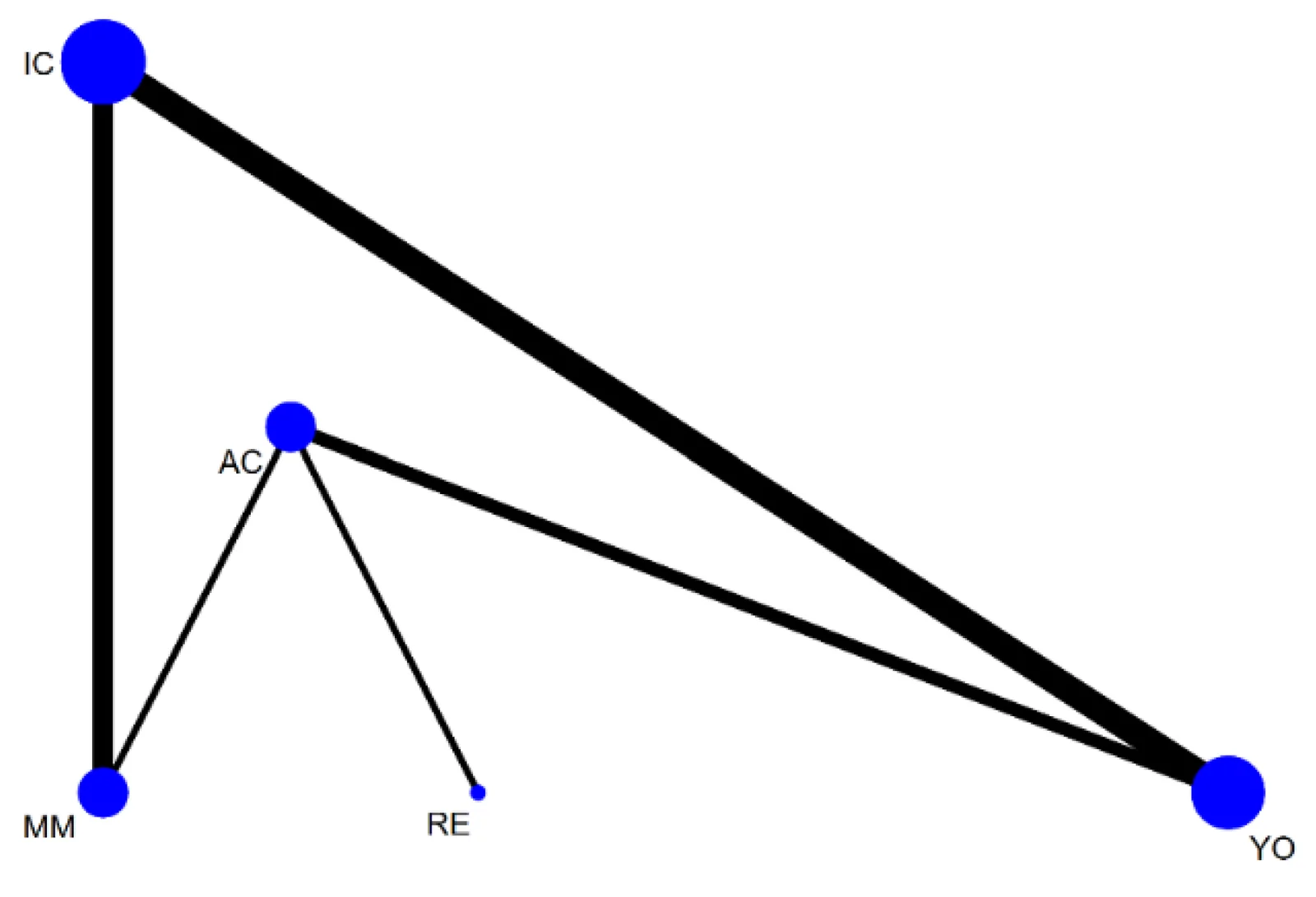
Network geometry of the strict exercise network. Nodes represent the five treatment categories included in the strict network, with node size proportional to the number of studies involving each treatment. Edge thickness is proportional to the number of randomized direct comparisons between treatment pairs. The strict network comprised 11 randomized direct comparisons across five treatment pairs, involving 754 participants. No direct head-to-head comparisons were available between the exercise modalities. AC, active control; IC, inactive control; MM, multimodal exercise; RE, resistance exercise; YO, yoga.

**Figure 4:**
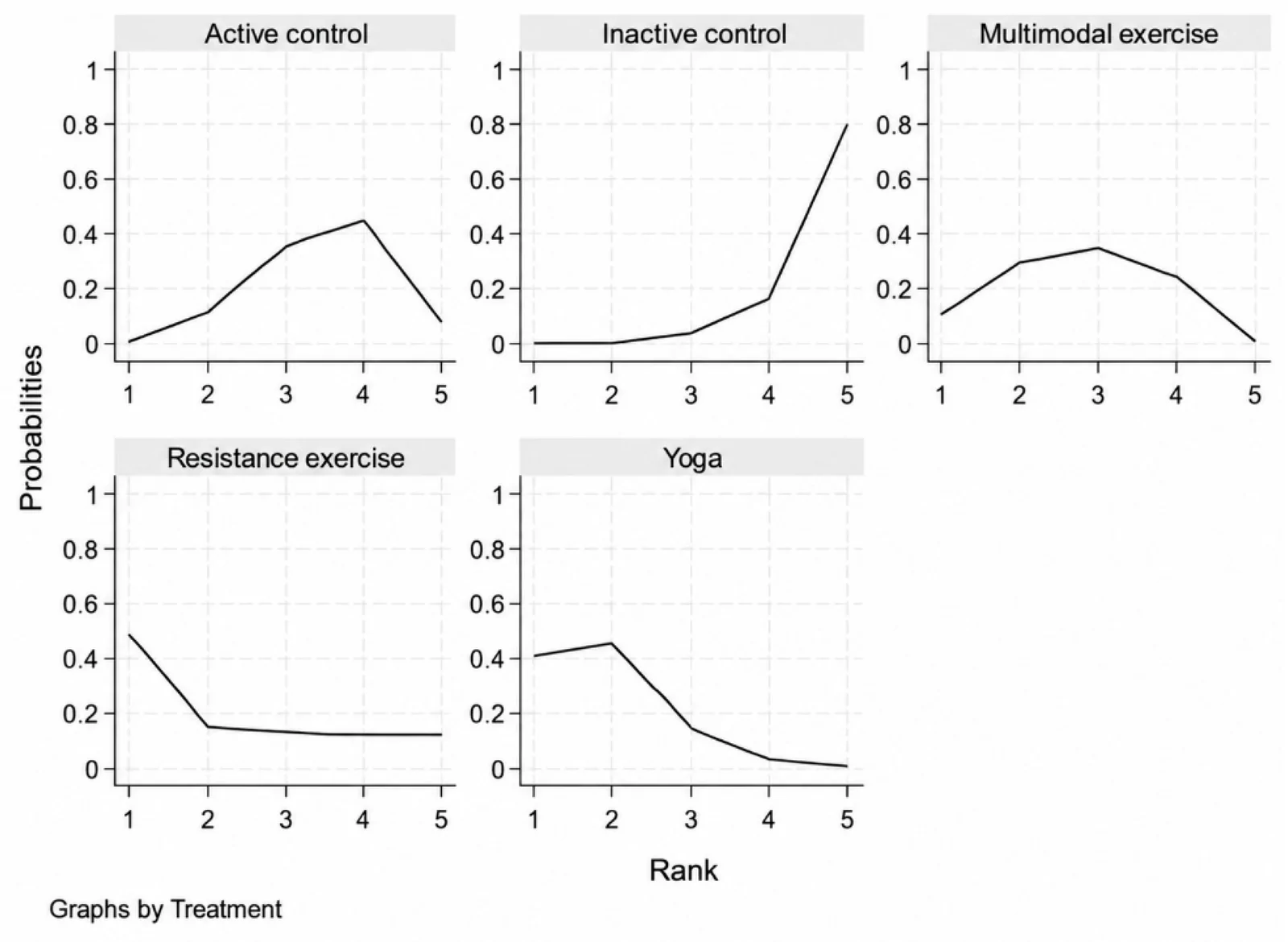
Rankograms for treatments in the strict exercise network. Each curve represents the probability of a treatment occupying each possible rank, with rank 1 indicating the most favorable treatment. Ranking probabilities were derived from the random-effects consistency model using 10,000 simulation draws (seed = 20,260,816). The distributions indicate uncertainty in the relative treatment hierarchy, particularly between yoga and resistance exercise.

**Table 1:** Characteristics of Studies Included in the Systematic Review.

Study	Population	Randomized N	Exercise or Movement-Based Intervention	Comparator	Intervention Exposure	PTSD Outcome	Assessment	Intervention Eligibility Framework
Carter et al. (2013) [41]	Male Vietnam veterans with chronic, treatment-resistant DSM-IV PTSD	31	Adapted Sudarshan Kriya Yoga breathing program	Waitlist	Intensive 5-day program followed by group sessions; randomized comparison assessed at 6 weeks	CAPS	Clinician-rated	Broad-only: intervention
Gelkopf et al. (2013) [45]	Veterans with chronic combat-related PTSD receiving rehabilitation services	42	Nature Adventure Rehabilitation centered on sailing	Waitlist	3 h group session weekly for 12 months	Modified 30-item SASRQ total PTS score	Self-report	Pairwise-only/recreation
Kim et al. (2013) [40]	Nurses with subclinical PTSD symptoms	22 ^2^	Mindfulness-based stretching and deep-breathing exercise	No intervention	8 weeks; 2 sessions/week; 60 min/session	PCL-C	Self-report	Broad-only: intervention ^2^
Mitchell et al. (2014) [27]	Veteran and civilian women with current full or subthreshold PTSD	38	Kripalu-based trauma-sensitive yoga	Assessment-only control	12 × 75 min sessions; delivered over 6 or 12 weeks	PCL-C	Self-report	Strict-eligible
Seppälä et al. (2014) [38]	Male veterans of the Iraq or Afghanistan wars with PTSD symptoms	21	Sudarshan Kriya breathing-based meditation	Waitlist/assessment control	3 h/day for 7 consecutive days	PCL-M	Self-report	Broad-only: intervention
van der Kolk et al. (2014) [30]	Women with chronic, treatment-resistant PTSD related to interpersonal trauma	64	Trauma-informed hatha yoga	Supportive women’s health education	10 weeks; 1 session/week; 60 min/session	CAPS	Clinician-rated	Strict-eligible
Jindani et al. (2015) [37]	Community adults screening positive for PTSD symptoms	80	Kundalini Yoga trauma protocol	Waitlist	8 weeks; 1 session/week; 90 min/session plus home practice	PCL-17	Self-report	Strict-eligible
Powers et al. (2015) [28]	Community adults with a principal DSM-IV diagnosis of PTSD	9	Prolonged exposure therapy augmented with moderate-intensity treadmill exercise	Prolonged exposure therapy alone	12 weeks; 12 weekly PE sessions; 30 min treadmill exercise at ~70% age-predicted HRmax immediately before each PE session	PSSI	Clinician-rated	Strict-eligible ^1^
Quinones et al. (2015) [31]	Reintegrating former combatants in Colombia with PTSD	100	Satyananda Yoga plus the regular reintegration program	Treatment as usual/reintegration program	16 weeks; 2 sessions/week; 60 min/session	PCL-C	Self-report	Strict-eligible
Rosenbaum et al. (2015) [29]	Inpatients with psychiatrist-confirmed primary DSM-IV-TR PTSD	81	Resistance training plus pedometer-based walking, in addition to usual care	Usual care	12 weeks; three 30 min resistance sessions/week plus walking	PCL-C	Self-report	Strict-eligible
Goldstein et al. (2018) [33]	Veterans with current or partial PTSD	47	Integrative exercise combining aerobic and resistance exercise with mindfulness-based practices	Waitlist	12 weeks; 3 sessions/week; 60 min/session	CAPS	Clinician-rated	Strict-eligible
Johnson et al. (2018) [42]	Veterans with PTSD and/or traumatic brain injury	29	Therapeutic horseback riding	Waitlist crossover	6 weeks; 1 session/week; 60 min/session	PCL-M	Self-report	Broad-only: intervention ^3^
Reinhardt et al. (2018) [32]	Military veterans and active-duty personnel with DSM-IV PTSD	51	Kripalu yoga	Assessment-only/no-treatment control	10 weeks; 2 sessions/week; 90 min/session	CAPS	Clinician-rated	Strict-eligible
Whitworth et al. (2019) [43]	Community adults screening positive for PTSD	22	High-intensity resistance training	Time-matched attention control	3 weeks; 3 sessions/week; 30 min/session	PDS-5	Self-report	Strict-eligible
Davis et al. (2020) [46]	Veterans and civilians with CAPS-5-confirmed PTSD; predominantly veterans	212	Holistic Hatha Yoga Program	Wellness Lifestyle Program including didactic discussion and 20 min group walking	16 weeks; 1 session/week; 90 min/session	CAPS-5	Clinician-rated	Strict-eligible
Huberty et al. (2020) [39]	Women with stillbirth-related probable PTSD symptoms	90 (30/30/30)	Low- or moderate-dose online yoga	Stretching and toning	12 weeks; self-directed dose of approximately 60 or 150 min/week	IES-R	Self-report	Strict-eligible
Young-McCaughan et al. (2022) [35]	Active-duty military personnel with PTSD	72	Four-arm factorial trial of aerobic exercise, imaginal exposure, their combination, and self-care	Self-care/psychotherapy comparator according to factorial contrast	8-week intervention	PCL-S	Self-report	Strict-eligible
Bryant et al. (2023) [34]	Adults with clinically diagnosed PTSD	130	Exposure therapy augmented with brief aerobic exercise	Exposure therapy plus passive stretching	10-week intervention	CAPS-2	Clinician-rated	Strict-eligible
Neylan et al. (2025) [36]	U.S. veterans with current PTSD and/or clinically relevant CAPS-5 symptoms	84	Integrative aerobic, resistance, stretching, and mindful body/breath exercise	PTSD Recovery Class psychoeducation	12 weeks; 3 sessions/week; 60 min/session	CAPS-5	Clinician-rated	Strict-eligible
Nilsson et al. (2026) [44]	Trauma-affected adult refugees with probable PTSD (PCL-5 ≥ 31)	183	Multicomponent trauma-informed physical-activity program	Waitlist with brief physical-activity information	10 sessions over approximately 10–12 weeks; 1–2 h/week	PCL-5	Self-report	Strict-eligible

Note. Intervention eligibility framework indicates whether the intervention satisfied the prespecified strict physical-exercise definition or was eligible only under the broad exercise and movement-based definition. Participant eligibility was common to both analytical frameworks and included adults with either a formal PTSD diagnosis or clinically significant PTSD symptoms established using a validated PTSD-specific measure. “Strict-eligible” indicates that the intervention met the structured physical-exercise definition, whereas “Broad-only: intervention” indicates eligibility only under the broad exercise and movement-based definition. “Pairwise-only/recreation” indicates an eligible intervention that contributed to the conventional pairwise analysis but could not be mapped to a prespecified clinically coherent network node. Eligibility classification does not imply that a study contributed to every statistical model; analytical inclusion additionally depended on the availability and compatibility of effect-size data. Randomized N is reported rather than a single “N analyzed” because the relevant analytical denominators varied according to outcome availability and estimand. ^1^ Powers et al. (2015) [28] satisfied the strict intervention and population eligibility criteria but did not contribute to the primary raw post-treatment meta-analysis or network meta-analysis because post-treatment arm-level standard deviations were unavailable. The reported between-group effect was based on pre–post change and was retained in the alternative-estimand audit to document the available randomized evidence but was not combined with the primary quantitative synthesis. ^2^ Kim et al. included an additional non-PTSD normative group in the publication; the randomized PTSD comparison comprised 11 intervention and 11 control participants. ^3^ Johnson et al. used a randomized waitlist crossover design; the published post-riding statistics pool participants across treatment periods, so a clean randomized-period treatment contrast is not directly recoverable from the reported summary statistics. Abbreviations: CAPS, Clinician-Administered PTSD Scale; CAPS-2, Clinician-Administered PTSD Scale-2; CAPS-5, Clinician-Administered PTSD Scale for DSM-5; DSM-IV, Diagnostic and Statistical Manual of Mental Disorders, Fourth Edition; DSM-IV-TR, Diagnostic and Statistical Manual of Mental Disorders, Fourth Edition, Text Revision; HRmax, maximum heart rate; IES-R, Impact of Event Scale–Revised; PCL-17, 17-item Posttraumatic Stress Disorder Checklist; PCL-5, PTSD Checklist for DSM-5; PCL-C, PTSD Checklist–Civilian Version; PCL-M, PTSD Checklist–Military Version; PCL-S, PTSD Checklist–Specific Version; PDS-5, Posttraumatic Diagnostic Scale for DSM-5; PE, prolonged exposure; PSSI, PTSD Symptom Scale–Interview Version; PTS, post-traumatic stress; PTSD, post-traumatic stress disorder; SASRQ, Stanford Acute Stress Reaction Questionnaire.

**Table 2:** Network Meta-analysis Relative Effects in the Strict Exercise Network.

Exercise Modality	Direct Studies, k	Evidence Basis	SMD vs. Active Control (95% CI)
Yoga	2	Direct + indirect	−0.32 (−0.73 to 0.08)
Multimodal	1	Direct + indirect	−0.13 (−0.63 to 0.37)
Resistance	1	Direct only	−0.32 (−1.32 to 0.68)

Note. Estimates are expressed as Hedges’ *g* standardized mean differences with 95% confidence intervals and were obtained from the random-effects consistency network meta-analysis. Negative values indicate greater reductions in PTSD symptoms relative to active control. Direct studies, k, denotes the number of randomized direct comparisons with active control. “Direct + indirect” indicates that the network estimate incorporated both direct evidence and indirect evidence through the available closed loop; “Direct only” indicates that no indirect evidence was available for that comparison.

**Table 3:** Treatment Ranking and CINeMA Confidence in Network Estimates.

Treatment	Probability of Ranking First (%)	Mean Rank	SUCRA	CINeMA Confidence vs. Active Control
Yoga	40.4	1.8	0.809	Very low
Resistance exercise	47.9	2.3	0.685	Very low
Multimodal exercise	10.6	2.7	0.563	Very low
Active control	1.1	3.5	0.383	Reference
Inactive control	0.0	4.8	0.059	Very low

Note. Ranking probabilities, mean ranks, and SUCRA values were derived from the random-effects consistency model using 10,000 simulation draws (seed = 20,260,816), with lower PTSD symptom scores indicating more favorable outcomes. CINeMA confidence ratings refer to the corresponding network effect estimates versus active control and not to the certainty of the ranking statistic itself. Confidence was assessed across within-study bias, reporting bias, indirectness, imprecision, heterogeneity, and incoherence.

## Data Availability

All data analyzed in this study were extracted from previously published reports. The study-level data and analysis datasets supporting the findings of this systematic review and meta-analysis are provided in Supplementary Dataset S1. No new individual-level participant data were generated.

## Supplementary Materials

The following supporting information can be downloaded at: https://www.mdpi.com/article/10.3390/healthcare14193166/s1, Table S1: Full Search Strategies and Additional Information Sources; Table S2: Full-Text Reports Excluded after Eligibility Assessment; Table S3: Detailed Data-extraction and Coding Audit; Table S4: Study-level Risk-of-Bias Assessment (RoB 2); Table S5: Full CINeMA Assessment of Confidence in the Network Meta-analysis Estimates; Table S6: Results of the Broad Network Meta-analysis Sensitivity Analysis; Table S7: Distribution of Potential Effect Modifiers Across Direct Comparisons in the Strict Exercise Network; Table S8: Alternative-Estimand Audit of Eligible Randomized Trials Not Included in the Primary Raw Post-Treatment Quantitative Synthesis; Figure S1: Risk-of-bias traffic-light plot for included randomized trials; Figure S2: Summary of risk-of-bias judgments across included randomized trials; Figure S3: Network geometry of the broad exercise and movement-based intervention network; Figure S4: Network relative effects in the broad exercise and movement-based intervention network; Figure S5: Rankograms for treatments in the broad exercise and movement-based intervention network; Figure S6: Comparison-adjusted funnel plot for the strict exercise network; Figure S7: Leave-one-study-out sensitivity analysis of the conventional pairwise meta-analysis; Figure S8: Subgroup and sensitivity analyses of the conventional pairwise meta-analysis; Figure S9: Funnel plot for the conventional pairwise meta-analysis. Dataset S1: Study-level data and analysis datasets used for the conventional pairwise meta-analysis, strict and broad network meta-analyses, alternative-estimand audit, CINeMA assessment, and risk-of-bias evaluation, including a variable codebook and quality-control checks.

## Abbreviations

The following abbreviations are used in this manuscript:

CINeMA	Confidence in Network Meta-Analysis
CI	Confidence Interval
DSM	Diagnostic and Statistical Manual of Mental Disorders
PRISMA	Preferred Reporting Items for Systematic Reviews and Meta-Analyses
PRISMA-NMA	Preferred Reporting Items for Systematic Reviews and Meta-Analyses Extension for Network Meta-Analyses
PROSPERO	International Prospective Register of Systematic Reviews
PTSD	Post-Traumatic Stress Disorder
REML	Restricted Maximum Likelihood
RoB 2	Cochrane Risk of Bias 2
SIDE	Separating Indirect from Direct Evidence
SMD	Standardized Mean Difference
SUCRA	Surface Under the Cumulative Ranking Curve
