# Supplementary material for: Exercise Interventions for PTSD: A Systematic Review and Network Meta-Analysis of Overall Effects, Comparative Effectiveness, and Ranking Uncertainty
Source: Healthcare (Basel). 2026 Sep 24;14(19):3166. doi: 10.3390/healthcare14193166 (PMC13665407; doi:10.3390/healthcare14193166)
Supplement: Supplementary file 1 [file healthcare-14-03166-s001.zip › (final)Supplementary.pdf]

[Supplementary Tables]

Supplementary Table S1. Full Search Strategies and Additional Information Sources

Database / source	Platform	Coverage	Full search strategy	Restrictions	Records retrieved
PubMed	National Library of Medicine	Database inception to 31 January 2026	("Stress Disorders, Post-Traumatic"[Mesh] OR "post-traumatic stress disorder"[Title/Abstract] OR "posttraumatic stress disorder"[Title/Abstract] OR PTSD[Title/Abstract]) AND ("Exercise"[Mesh] OR "Exercise Therapy"[Mesh] OR "Motor Activity"[Mesh] OR "Resistance Training"[Mesh] OR "Yoga"[Mesh] OR exercise[Title/Abstract] OR "physical activity"[Title/Abstract] OR "exercise training"[Title/Abstract] OR "aerobic exercise"[Title/Abstract] OR "aerobic training"[Title/Abstract] OR "resistance exercise"[Title/Abstract] OR "resistance training"[Title/Abstract] OR "strength training"[Title/Abstract] OR walking[Title/Abstract] OR running[Title/Abstract] OR yoga[Title/Abstract] OR "mind-body"[Title/Abstract] OR stretching[Title/Abstract] OR breathing[Title/Abstract] OR meditation[Title/Abstract] OR equine[Title/Abstract] OR horse*[Title/Abstract]) AND (randomized controlled trial[Publication Type] OR controlled clinical trial[Publication Type] OR random*[Title/Abstract] OR trial[Title/Abstract]) NOT (animals[MeSH Terms] NOT humans[MeSH Terms])	No lower publication-date limit; search coverage ended on 31 January 2026. English-language eligibility applied during screening; non-human-only records were excluded using the stated filter.	494
APA PsycINFO	EBSCOhost	Database inception to 31 January 2026	(TI ("post-traumatic stress disorder" OR "posttraumatic stress disorder" OR PTSD) OR AB ("post-traumatic stress disorder" OR "posttraumatic stress disorder" OR PTSD)) AND (TI (exercise OR "physical activity" OR "exercise training" OR "aerobic exercise" OR "aerobic training" OR "resistance exercise" OR "resistance training" OR "strength training" OR walking OR running OR yoga OR "mind-body" OR stretching OR breathing OR meditation OR equine OR horse*) OR AB (exercise OR "physical activity" OR "exercise training" OR "aerobic exercise" OR "aerobic training" OR "resistance exercise" OR "resistance training" OR "strength training" OR walking OR running OR yoga OR "mind-body" OR stretching OR breathing OR meditation OR equine OR horse*)) AND (TI (random* OR trial OR controlled) OR AB (random* OR trial OR controlled))	No publication-year restriction; English-language eligibility applied during screening	6,627
Cochrane Central Register of Controlled Trials (CENTRAL)	Cochrane Library	Database inception to 31 January 2026	#1 MeSH descriptor: [Stress Disorders, Post-Traumatic] explode all trees; #2 ("post-traumatic stress disorder" OR "posttraumatic stress disorder" OR PTSD):ti,ab,kw; #3 #1 OR #2; #4 MeSH descriptor: [Exercise] explode all trees; #5 MeSH descriptor: [Exercise Therapy] explode all trees; #6 MeSH descriptor: [Motor Activity] explode all trees; #7 MeSH descriptor: [Resistance Training] explode all trees; #8 MeSH descriptor: [Yoga] explode all trees; #9 (exercise OR "physical activity" OR "exercise training" OR "aerobic exercise" OR "aerobic training" OR "resistance exercise" OR "resistance training" OR "strength training" OR walking OR running OR yoga OR "mind-body" OR stretching OR breath* OR meditation OR equine OR horse*):ti,ab,kw; #10 #4 OR #5 OR #6 OR #7 OR #8 OR #9; #11 #3 AND #10	No additional RCT filter applied because CENTRAL is a trials-focused database; no publication-year restriction	963

Database / source	Platform	Coverage	Full search strategy	Restrictions	Records retrieved
ScienceDirect	Elsevier ScienceDirect	Database inception to 31 January 2026	Search 1:<br>("post-traumatic stress disorder" OR "posttraumatic stress disorder" OR PTSD) AND (exercise OR "physical activity" OR yoga) AND (randomized OR randomised OR trial)<br><br>Search 2:<br>("post-traumatic stress disorder" OR PTSD) AND ("aerobic exercise" OR "resistance training" OR walking OR running) AND (randomized OR trial)<br><br>Search 3:<br>("post-traumatic stress disorder" OR PTSD) AND (stretching OR "mind-body" OR meditation OR breathing) AND (randomized OR trial)<br><br>Search 4:<br>("post-traumatic stress disorder" OR PTSD) AND (equine OR horse) AND (randomized OR trial)	Search conducted in title, abstract, and author-specified keywords where supported; research articles; no publication-year restriction; English-language eligibility applied during screening	29,459
Other sources	Manual backward citation searching	Not applicable	Reference lists of included randomized trials and relevant systematic reviews were manually examined to identify potentially eligible reports not retrieved through the electronic database searches.	Studies were assessed using the same prespecified eligibility criteria as electronically identified records	0

Note. Searches were conducted from database inception to the date indicated for each source, with no restriction on publication year. Search strategies combined controlled vocabulary, where supported, with free-text terms representing post-traumatic stress disorder and exercise or movement-based interventions. Randomized-trial terms were applied in bibliographic databases where appropriate; no additional randomized-trial filter was applied to CENTRAL. English-language eligibility was assessed according to the prespecified eligibility criteria. Reference lists of included trials and relevant systematic reviews were manually screened for additional potentially eligible reports. PTSD, post-traumatic stress disorder; RCT, randomized controlled trial; CENTRAL, Cochrane Central Register of Controlled Trials; The final database searches were conducted on 7 February 2026. Records available through 31 January 2026 were eligible for inclusion.

Supplementary Table S2. Full-Text Reports Excluded after Eligibility Assessment

No.	Study	Report title	Primary reason for exclusion	Detailed justification
1	Dick et al. (2014)	Examining Mechanisms of Change in a Yoga Intervention for Women: The Influence of Mindfulness, Psychological Flexibility, and Emotion Regulation on PTSD Symptoms	Duplicate/secondary report	This report presented a secondary mechanistic analysis of the same randomized cohort reported in Mitchell et al. (2014). The analysis included 38 women with full or subthreshold PTSD from the yoga-versus-assessment-control trial and was excluded as a separate study to prevent double counting of the same randomized participants.
2	Possemato et al. (2016)	A Randomized Clinical Trial of Primary Care Brief Mindfulness Training for Veterans with PTSD	Ineligible intervention	The intervention was a four-session brief mindfulness program incorporating sitting meditation, body scan, moving meditation, gentle yoga, and walking meditation. Although movement-based practices were present, they were components of a broader mindfulness intervention rather than the primary therapeutic component, and the independent contribution of physical exercise or movement could not be distinguished. The intervention therefore did not satisfy the prespecified strict or broad exercise/movement eligibility criteria.
3	Polusny et al. (2015)	Mindfulness-Based Stress Reduction for Posttraumatic Stress Disorder Among Veterans: A Randomized Clinical Trial	Ineligible intervention	The intervention was a multicomponent mindfulness-based stress reduction program in which mindfulness meditation constituted the principal therapeutic framework. Although movement- or yoga-related practices formed part of the program, they were not evaluated as a distinguishable primary exercise or movement intervention. The study therefore did not meet the prespecified intervention eligibility criteria.
4	Kelly and Garland (2016)	Trauma-Informed Mindfulness-Based Stress Reduction for Female Survivors of Interpersonal Violence: Results from a Stage I RCT	Ineligible intervention	The trauma-informed mindfulness-based stress reduction program combined guided meditation, gentle movement exercises, didactic instruction, and group discussion. Movement was an ancillary component of a broader mindfulness-based psychological intervention rather than a distinguishable primary exercise or movement treatment; therefore, the intervention did not satisfy the prespecified eligibility criteria.

No.	Study	Report title	Primary reason for exclusion	Detailed justification
5	Shivakumar et al. (2017)	Exercise for PTSD in Women Veterans: A Proof-of-Concept Study	Ineligible study design	This proof-of-concept study evaluated a 12-week moderate-intensity exercise program but did not include a concurrent randomized comparator group. The investigators explicitly noted that the study could not determine the unique contribution of exercise and that randomized controlled studies were required. Therefore, a valid randomized between-group treatment effect could not be estimated.
6	Whitworth et al. (2023)	Acute Changes in Affective Valence and Perceived Distress Predict Reductions in PTSD Symptom Severity	Overlapping/secondary analysis	This report pooled participant data from two randomized controlled trials, including participants from the Whitworth et al. (2019) trial already represented in the review and an additional replication sample. Because an independent effect for the nonoverlapping replication cohort could not be isolated reliably from the pooled report, the article was excluded as a separate study to prevent participant overlap and unit-of-analysis error.
7	Newman and Motta (2007)	The Effects of Aerobic Exercise on Childhood PTSD, Anxiety, and Depression	Ineligible population	The study was conducted exclusively in adolescent girls with childhood PTSD, whereas the prespecified eligibility criteria for the present review were restricted to adults. The report was therefore excluded on the basis of population eligibility.

Note. A single primary reason for exclusion was assigned to each full-text report in accordance with PRISMA reporting principles, even when more than one eligibility concern was present. Secondary publications and overlapping samples were excluded as independent studies to maintain statistical independence and prevent double counting. Multicomponent mindfulness interventions were considered ineligible when movement or exercise was only an ancillary component and its independent therapeutic contribution could not be distinguished from the broader intervention. PTSD, post-traumatic stress disorder; RCT, randomized controlled trial.

Supplementary Table S3. Detailed Data-extraction and Coding Audit

No.	Study	Randomized N	Selected analysis N (I/C)	Intervention classification	Comparator classification	PTSD outcome	Outcome assessor	Effect basis	Primary pairwise	Strict NMA	Broad NMA	Alternative-estimand audit	Coding and verification note
1	Carter 2013	31	14/11	Breathing-meditation	Inactive control	CAPS	Clinician-administered	Raw post-treatment arm data	Yes	No	Yes	No	First randomized 6-week period only.
2	Gelkopf 2013	42	22/20	Adventure/recreation	Inactive control	Modified SASRQ PTS	Self-report	Raw post-treatment arm data	Yes	No	No	No	Pairwise only; intervention could not be mapped to a prespecified coherent NMA node.
3	Kim 2013	22	11/11	Mindful stretching	Inactive control	PCL-C	Self-report	Raw post-treatment arm data	Yes	No	Yes	No	Broad-only intervention under the prespecified manuscript definition.
4	Mitchell 2014	38	20/18	Yoga	Inactive control	PCL-C	Self-report	Raw post-treatment arm data	Yes	Yes	Yes	No	Raw-post primary and NMA eligible.
5	Seppälä 2014	21	10/10	Breathing-meditation	Inactive control	PCL-M	Self-report	Raw post-treatment arm data	Yes	No	Yes	No	Broad-only intervention.
6	van der Kolk 2014	64	31/29	Yoga	Active control	CAPS	Clinician-administered	Raw post-treatment arm data	Yes	Yes	Yes	No	Women's health education classified as active control.
7	Jindani 2015	80	29/21	Yoga	Inactive control	PCL-17	Self-report	Raw post-treatment arm data	Yes	Yes	Yes	No	Symptom-threshold population retained under the current participant eligibility criteria.

No.	Study	Randomized N	Selected analysis N (I/C)	Intervention classification	Comparator classification	PTSD outcome	Outcome assessor	Effect basis	Primary pairwise	Strict NMA	Broad NMA	Alternative-estimand audit	Coding and verification note
8	Powers 2015	9	NR	Aerobic exercise augmentation	Active control	PSSI	Clinician-administered/interview	Reported change-score SMD	No	No	No	Yes	Randomized PE+exercise versus PE alone trial; controlled change-score d=2.65, SE=0.92. Raw post-treatment arm SDs were unavailable, so the study was retained in the coding audit as an alternative estimand rather than being combined with the primary raw post-treatment analysis.
9	Quinones 2015	100	48/50	Yoga	Inactive control	PCL-C	Self-report	Raw post-treatment arm data	Yes	Yes	Yes	No	TAU/reintegration care classified as inactive control.
10	Rosenbaum 2015	81	30/28	Multimodal exercise	Inactive control	PCL-C	Self-report	Raw post-treatment arm data	Yes	Yes	Yes	No	Resistance plus walking intervention; usual care classified as inactive control.
11	Goldstein 2018	47	21/26	Multimodal exercise	Inactive control	CAPS	Clinician-administered	Raw post-treatment arm data	Yes	Yes	Yes	No	Observed post-treatment values used; original article reported longitudinal statistics on an ITT/all-available-data basis.
12	Johnson 2018	29	NR	Equine-assisted movement	Inactive control	PCL-M	Self-report	Crossover pooled data	No	No	No	No	Published riding statistics pooled crossover periods; a clean independent randomized contrast could not be reconstructed.
13	Reinhardt 2018	51	9/6	Yoga	Inactive control	CAPS	Clinician-administered	Raw post-treatment arm data	Yes	Yes	Yes	No	Completer outcome used; substantial attrition was documented.
14	Whitworth 2019	22	9/10	Resistance exercise	Active control	PDS-5	Self-report	Raw post-treatment arm data	Yes	Yes	Yes	No	Analysis denominator verified against the trial flow information (9/10).

No.	Study	Randomized N	Selected analysis N (I/C)	Intervention classification	Comparator classification	PTSD outcome	Outcome assessor	Effect basis	Primary pairwise	Strict NMA	Broad NMA	Alternative-estimand audit	Coding and verification note
15	Davis 2020	212	70/70	Yoga	Active control	CAPS-5	Clinician-administered	Raw post-treatment arm data	Yes	Yes	Yes	No	Wellness plus walking comparator classified as active control.
16	Huberty 2020	90	NR	Yoga (dose-ranging)	Active control	IES-R	Self-report	Adjusted model estimate	No	No	No	Yes	Adjusted model-based estimates from the three-arm trial were reported; raw post-treatment SDs and exact outcome-specific denominators were unavailable. The reported estimates were therefore retained in the alternative-estimand audit but were not combined with the primary raw post-treatment analyses.
17	Young-McCaughan 2022	72	8/11	Aerobic exercise	Active control	PCL-S	Self-report	Model-based estimate	No	No	No	Yes	Four-arm factorial trial; estimated marginal means and SEs were available, but raw post-treatment SD and covariance information was insufficient to reconstruct the primary raw post-treatment estimand. The model-based estimate was therefore retained in the alternative-estimand audit but was not combined with the primary quantitative synthesis.
18	Bryant 2023	130	58/54	Aerobic exercise augmentation	Active control	CAPS-2	Clinician-administered	Model-based estimate	No	No	No	Yes	Model-estimated effect retained in the alternative-estimand audit but not combined with the primary raw post-treatment dataset.
19	Neylan 2025	84	29/34	Multimodal exercise	Active control	CAPS-5	Clinician-administered	Raw post-treatment arm data	Yes	Yes	Yes	No	Recovery psychoeducation classified as active control.

No.	Study	Randomized N	Selected analysis N (I/C)	Intervention classification	Comparator classification	PTSD outcome	Outcome assessor	Effect basis	Primary pairwise	Strict NMA	Broad NMA	Alternative-estimand audit	Coding and verification note
20	Nilsson 2026	183	86/80	Multimodal exercise	Inactive control	PCL-5	Self-report	Raw post-treatment arm data	Yes	Yes	Yes	No	Symptom-threshold population retained under the current participant eligibility criteria.

Note. Randomized N denotes the total number of participants randomized in the original trial, whereas selected analysis N denotes the intervention/control sample sizes contributing to the immediate post-treatment effect selected for the corresponding quantitative analysis. Primary pairwise, strict NMA, and broad NMA indicate whether each study contributed to the respective analytical dataset. Studies for which a comparable raw immediate post-treatment arm-level effect could not be reconstructed were retained in the systematic review and coding audit but were not combined with the primary raw post-treatment estimand. Model-based, change-score, or other alternative effect estimates were documented separately in the alternative-estimand audit to preserve transparency regarding the available randomized evidence and the basis for quantitative-analysis eligibility. I/C, intervention/control; NMA, network meta-analysis; NR, not reliably available.

Supplementary Table S4. Study-level Risk-of-Bias Assessment (RoB 2)

Study	D1	D2	D3	D4	D5	Overall
Bryant 2023	SC	SC	Low	Low	Low	Some concerns
Young-McCaughan 2022	SC	SC	Low	SC	SC	Some concerns
Neylan 2025	Low	Low	Low	Low	Low	Low
Huberty 2020	Low	SC	Low	SC	Low	Some concerns
Quinones 2015	Low	SC	Low	SC	SC	Some concerns
van der Kolk 2014	Low	Low	Low	Low	Low	Low
Goldstein 2018	Low	Low	Low	Low	Low	Low
Nilsson 2026	Low	Low	Low	SC	Low	Some concerns
Kim 2013	SC	SC	SC	SC	SC	Some concerns
Rosenbaum 2015	Low	Low	Low	SC	Low	Some concerns
Whitworth 2019	Low	SC	Low	SC	SC	Some concerns
Jindani 2015	Low	SC	High	SC	SC	High
Powers (2015)	SC	SC	Low	Low	SC	Some concerns
Mitchell 2014	SC	Low	Low	SC	SC	Some concerns
Reinhardt 2018	Low	SC	High	Low	SC	High
Carter 2013	Low	Low	SC	Low	SC	Some concerns
Seppälä 2014	Low	Low	Low	SC	SC	Some concerns
Johnson 2018	SC	SC	High	SC	SC	High
Gelkopf 2013	SC	SC	High	SC	SC	High
Davis 2020	Low	Low	High	Low	SC	High

D1= randomization process; D2= deviations from intended interventions; D3= missing outcome data; D4= measurement of the outcome; D5= selection of the reported result; SC= some concerns.

Supplementary Table S5. Full CINeMA Assessment of Confidence in the Network Meta-analysis Estimates

**Network comparison**	**Within-study bias**	**Reporting bias**	**Indirectness**	**Imprecision**	**Heterogeneity**	**Incoherence**	**Overall CINeMA confidence**
Active control vs Multimodal exercise	Some concerns	Some concerns	No concerns	Major concerns	No concerns	No concerns	Very low
Active control vs Resistance exercise	Some concerns	Some concerns	No concerns	Major concerns	No concerns	No concerns	Very low
Active control vs Yoga	Some concerns	Some concerns	No concerns	Some concerns	Some concerns	No concerns	Very low
Active control vs Inactive control	Some concerns	Some concerns	No concerns	Some concerns	Some concerns	No concerns	Very low
Inactive control vs Multimodal exercise	Some concerns	Some concerns	No concerns	No concerns	Major concerns	No concerns	Very low
Inactive control vs Yoga	Some concerns	Some concerns	No concerns	No concerns	Some concerns	No concerns	Very low
Inactive control vs Resistance exercise	Some concerns	Some concerns	No concerns	Major concerns	No concerns	No concerns	Very low
Multimodal exercise vs Resistance exercise	Some concerns	Some concerns	No concerns	Major concerns	No concerns	No concerns	Very low
Multimodal exercise vs Yoga	Some concerns	Some concerns	No concerns	Major concerns	No concerns	No concerns	Very low
Resistance exercise vs Yoga	Some concerns	Some concerns	No concerns	Major concerns	No concerns	No concerns	Very low

Note. Confidence in network estimates was assessed using the Confidence in Network Meta-Analysis (CINeMA) framework across six domains: within-study bias, reporting bias, indirectness, imprecision, heterogeneity, and incoherence. An absolute SMD of 0.20 was used as a common standardized decision threshold for CINeMA judgments rather than as a validated PTSD-specific minimal clinically important difference. Within-study bias and indirectness were summarized using the average risk-of-bias and average indirectness approaches, respectively. The global design-by-treatment interaction test did not indicate statistically significant evidence of inconsistency ($\chi^2(1) = 2.35$, $P = .125$). SIDE analyses for the four comparisons supported by both direct and indirect evidence likewise showed no statistically significant direct–indirect disagreement (all $P = .125$). Comparisons informed by only one source of evidence were not evaluable using SIDE. In accordance with CINeMA guidance, the incoherence domain was rated as no concerns when SIDE P values exceeded .10 for comparisons supported by both direct and indirect evidence or when the global inconsistency test P value exceeded .10 for comparisons informed by only one source of evidence. Overall confidence ratings integrated judgments across all six domains. CINeMA, Confidence in Network Meta-Analysis; SIDE, separating indirect from direct evidence; SMD, standardized mean difference.

Supplementary Table S6. Results of the Broad Network Meta-analysis Sensitivity Analysis

Panel A. Network relative effects versus active control

Treatment	Evidence basis	SMD vs active control (95% CI)	*P*
Breathing-meditation	Indirect only	−0.34 (−1.15 to 0.48)	.419
Inactive control	Indirect only	0.32 (−0.15 to 0.78)	.181
Multimodal exercise	Direct + indirect	−0.13 (−0.61 to 0.35)	.598
Mindful stretching	Indirect only	−1.05 (−2.17 to 0.07)	.066
Resistance exercise	Direct only	−0.32 (−1.30 to 0.66)	.522
Yoga	Direct + indirect	−0.32 (−0.71 to 0.07)	.104

Note. Negative values indicate greater PTSD symptom reduction relative to active control.

Panel B. Treatment ranking in the broad network

Treatment	PrBest (%)	Mean rank	SUCRA
Mindful stretching	75.7	1.5	0.922
Yoga	2.8	3.2	0.638
Breathing-meditation	7.7	3.4	0.604
Resistance exercise	13.3	3.6	0.566
Multimodal exercise	0.5	4.4	0.433
Active control	0.0	5.2	0.293
Inactive control	0.0	6.7	0.044

Note. Rank 1 indicates the most favorable treatment. Ranking probabilities were estimated using 10,000 simulation draws (seed = 20260816), with lower PTSD symptom scores considered more favorable.

Panel C. Global and Local Inconsistency Assessment

Assessment	Comparison	Difference between direct and indirect estimates	*P* value
Global design-by-treatment inconsistency	Overall network	$\chi^2(1) = 2.57$	.109
SIDE	Active control vs Multimodal exercise	0.70	.109
SIDE	Active control vs Yoga	−0.70	.109
SIDE	Inactive control vs Multimodal exercise	−0.70	.109
SIDE	Inactive control vs Yoga	0.70	.109

Note. Global inconsistency was assessed using a design-by-treatment interaction model. Local incoherence was evaluated using separating indirect from direct evidence (SIDE) analyses for comparisons supported by both direct and indirect evidence. Breathing-meditation versus inactive control and mindful stretching versus inactive control were not considered evaluable SIDE comparisons because these leaf-node contrasts lacked an independent indirect pathway.

Supplementary Table S7. Distribution of Potential Effect Modifiers Across Direct Comparisons in the Strict Exercise Network

Direct comparison	Studies (k)	PTSD eligibility / clinical status	Population / trauma / age-sex profile	Intervention exposure	Comparator and concomitant care	PTSD outcome / assessor	Transitivity considerations
Yoga vs inactive control	Mitchell 2014; Jindani 2015; Quinones 2015; Reinhardt 2018 (k=4)	Current full or subthreshold PTSD; symptom-screen positive/current PTSD symptoms; PTSD in former combatants; DSM-IV PTSD	Veteran/civilian women; community adults; former combatants in Colombia; military veterans/active-duty personnel. One trial was women-only; age/sex distributions otherwise varied and were not consistently harmonized.	6–16 weeks; generally 1–2 supervised sessions/week; 75–90 min/session where reported; home practice included in Jindani.	Assessment-only, waitlist, or no-treatment comparators; Quinones included the regular reintegration/TAU program in both groups and was classified as inactive under the prespecified comparator rule.	PCL-C/PCL-17 self-report (3 studies); CAPS clinician-administered (1 study)	Marked variation in PTSD eligibility, military/civilian status, trauma context, intervention dose, and background care.
Yoga vs active control	van der Kolk 2014; Davis 2020 (k=2)	Chronic treatment-resistant PTSD related to interpersonal trauma; CAPS-5-confirmed PTSD	Women with chronic interpersonal-trauma PTSD versus a veteran/civilian PTSD sample. van der Kolk was women-only; age/sex distributions were not harmonized across the two trials.	10–16 weeks; 1 session/week; 60–90 min/session	Supportive women's health education; Wellness Lifestyle Program including didactic content and group walking.	CAPS/CAPS-5; clinician-administered (2 studies)	Common clinician-rated assessment, but substantial differences in trauma type, population composition, and active-comparator content.
Multimodal exercise vs inactive control	Rosenbaum 2015; Goldstein 2018; Nilsson 2026 (k=3)	Psychiatrist-confirmed primary DSM-IV-TR PTSD; current or partial PTSD; probable PTSD (PCL-5 ≥31)	Psychiatric inpatients; veterans; trauma-affected refugees. Rosenbaum reported mean age 47.8 years and 84% male; demographic distributions differed across studies and were not fully harmonized.	Approximately 10–12 weeks; multimodal programs varied in aerobic, resistance, walking, mindfulness, stretching, and/or trauma-informed physical-activity components.	Usual care in Rosenbaum (including psychotherapy, pharmacotherapy, and group therapy); waitlist in Goldstein; waitlist with brief physical-activity information in Nilsson.	PCL-C/PCL-5 self-report (2 studies); CAPS clinician-administered (1 study)	Heterogeneity in treatment setting, trauma/population profile, comparator content, concomitant care, and intervention components may modify relative effects.

Direct comparison	Studies (k)	PTSD eligibility / clinical status	Population / trauma / age-sex profile	Intervention exposure	Comparator and concomitant care	PTSD outcome / assessor	Transitivity considerations
Multimodal exercise vs active control	Neylan 2025 (k=1)	Current DSM-5 PTSD and/or clinically relevant symptoms (CAPS-5 ≥23)	U.S. veterans; age/sex characteristics were reported in the trial but could not be meaningfully compared within this single direct-comparison set.	12 weeks; 3 sessions/week; 60 min/session	PTSD Recovery Class psychoeducation (active comparator); other concomitant treatment was not standardized for cross-comparison in the review dataset.	CAPS-5; clinician-administered	Single-trial comparison; the distribution of effect modifiers within this direct-comparison set cannot be evaluated empirically.
Resistance exercise vs active control	Whitworth 2019 (k=1)	Community adults screening positive for PTSD symptoms	Community sample with heterogeneous trauma exposure; participants were predominantly women (81.8% in each randomized arm) and mean age was approximately 32 years.	3 weeks; 3 sessions/week; 30 min/session; high-intensity resistance training	Time-matched attention control; concomitant psychological/pharmacological treatment was not consistently characterized for cross-comparison.	PDS-5; self-report	Single small trial with a short intervention duration; the distribution of effect modifiers within this comparison cannot be assessed.
Overall strict network	11 randomized direct comparisons; 5 observed treatment-pair comparisons	Included clinically diagnosed PTSD, partial/subthreshold PTSD, and validated symptom-threshold populations.	Adult samples included military/veteran, civilian/community, inpatient, former-combatant, and refugee populations; trauma characteristics and sex composition varied substantially.	3–16 weeks across studies, with substantial variation in frequency, session duration, and intervention components.	7 comparisons involved inactive controls and 4 involved active controls; comparator intensity and concomitant care varied across studies.	6 self-report and 5 clinician-administered outcomes	Transitivity was considered plausible but uncertain. Sparse direct-comparison sets and incomplete/heterogeneous reporting precluded reliable formal comparison of effect-modifier distributions.

Note. Potential effect modifiers were summarized at the level of observed direct-comparison sets in the strict exercise network. PTSD eligibility/clinical status reflects diagnostic versus symptom-threshold entry criteria rather than a harmonized numeric baseline severity because PTSD measures and reporting formats differed across studies. Age, sex, trauma characteristics, and concomitant psychological or pharmacological treatment were summarized where sufficiently reported; incomplete and heterogeneous reporting precluded a reliable quantitative comparison of these characteristics across direct comparisons. No formal statistical tests of effect-modifier distributions were performed because several direct comparisons were represented by only one or a small number of trials. Comparator classifications follow the prespecified manuscript definitions. CAPS, Clinician-Administered PTSD Scale; PCL, PTSD Checklist; PDS-5, Posttraumatic Diagnostic Scale for DSM-5; PTSD, post-traumatic stress disorder; TAU, treatment as usual.

Supplementary Table S8. Alternative-Estimand Audit of Eligible Randomized Trials Not Included in the Primary Raw Post-Treatment Quantitative Synthesis

**Study**	**Randomized comparison relevant to exercise**	**Available estimand / time point**	**Available PTSD estimate**	**Direction of evidence**	**Why not pooled with primary raw-post Hedges' g**	**Sensitivity interpretation**
Powers et al. (2015)	Prolonged exposure + moderate-intensity aerobic exercise vs prolonged exposure alone	Controlled between-group standardized pre–post change; 12 weeks	Reported d = 2.65 (SE = 0.92) favoring PE + exercise. Post-treatment PSSI means were 5.20 (PE + exercise) and 8.25 (PE), but arm-level post-treatment SDs were not reported.	Favorable to exercise augmentation. The trial reported greater improvement in PTSD symptoms with PE + exercise.	The available effect is change-based rather than a raw post-treatment SMD, and post-treatment arm SDs were unavailable; therefore a compatible raw-post Hedges' g could not be reconstructed.	Directionally supports an exercise-augmentation benefit, but the estimand is not commensurate with the prespecified primary raw-post estimand.
Bryant et al. (2023; online 2022)	Exposure therapy + brief aerobic exercise vs exposure therapy + passive stretching	Least-square/model-estimated post-treatment means; 10 weeks	CAPS-2 adjusted means: 31.4 (95% CI 25.6–37.3) with exercise vs 35.3 (29.3–41.3) with passive stretching. Lower scores are favorable.	Numerically favorable to exercise augmentation at immediate post-treatment. The trial's primary 6-month endpoint also favored exercise augmentation.	Only adjusted/model-estimated means and CIs were available for the immediate post-treatment outcome; raw arm SDs and the covariance needed for a directly comparable raw-post SMD were unavailable.	Immediate post-treatment direction is consistent with benefit, but the available adjusted estimand cannot be pooled without additional assumptions.

Study	Randomized comparison relevant to exercise	Available estimand / time point	Available PTSD estimate	Direction of evidence	Why not pooled with primary raw-post Hedges' g	Sensitivity interpretation
Young-McCaughan et al. (2022)	Exercise only; imaginal exposure only; imaginal exposure + exercise; self-care control	Estimated marginal means with model SEs; 8 weeks	PCL-S means (SE): exercise 36.2 (4.28), imaginal exposure 34.4 (4.04), combined 34.3 (3.96), self-care 40.7 (3.86). Lower scores are favorable.	Numerically favorable for exercise-only and combined treatment vs self-care, but the combined arm was essentially similar to imaginal exposure alone. The trial reported no significant between-condition differences.	Model SEs are not arm-level SDs, and the four-arm model-based covariance structure was unavailable; therefore valid raw-post Hedges' g contrasts could not be reconstructed.	Provides no clear evidence that exercise, or exercise augmentation, was superior; results are compatible with a neutral comparative conclusion despite some favorable numerical differences.
Huberty et al. (2020)	Low-dose online yoga; moderate-dose online yoga; stretch-and-tone active control	Adjusted post-treatment means with 95% CIs; 12 weeks	IES-R adjusted means: low-dose yoga 24.53 (95% CI 16.72–32.34), moderate-dose yoga 20.38 (13.31–27.45), control 35.67 (26.22–45.12). Lower scores are favorable.	Numerically favorable to both yoga groups. However, the study reported no significant intervention-group effect or group-by-time interaction for PTSD and was not powered for efficacy.	Raw post-treatment SDs and exact outcome-specific arm denominators were not available, and adjusted-arm covariance was not reported; a compatible raw-post Hedges' g could not be reconstructed.	Directionally favorable but statistically inconclusive; supports retaining the study in the evidence audit rather than combining it with the primary raw-post synthesis.

Note. The primary quantitative synthesis used a prespecified common estimand based on compatible raw immediate post-treatment arm-level n, mean, and SD values converted to Hedges' g. The four trials above were otherwise eligible randomized studies but reported controlled change effects or adjusted/model-based post-treatment estimates that could not be converted to the primary estimand without additional unverified assumptions. Accordingly, they were retained in a structured alternative-estimand audit and were not statistically pooled with the primary raw-post synthesis. Directional interpretation refers to PTSD symptom severity at or nearest the immediate post-treatment time point; lower symptom scores indicate more favorable outcomes.

Abbreviations. CAPS-2, Clinician-Administered PTSD Scale; CI, confidence interval; IES-R, Impact of Event Scale-Revised; PE, prolonged exposure; PCL-S, PTSD Checklist-Stressor-Specific Version; PSSI, PTSD Symptom Scale-Interview; SD, standard deviation; SE, standard error; SMD, standardized mean difference.

[Supplementary Figures]

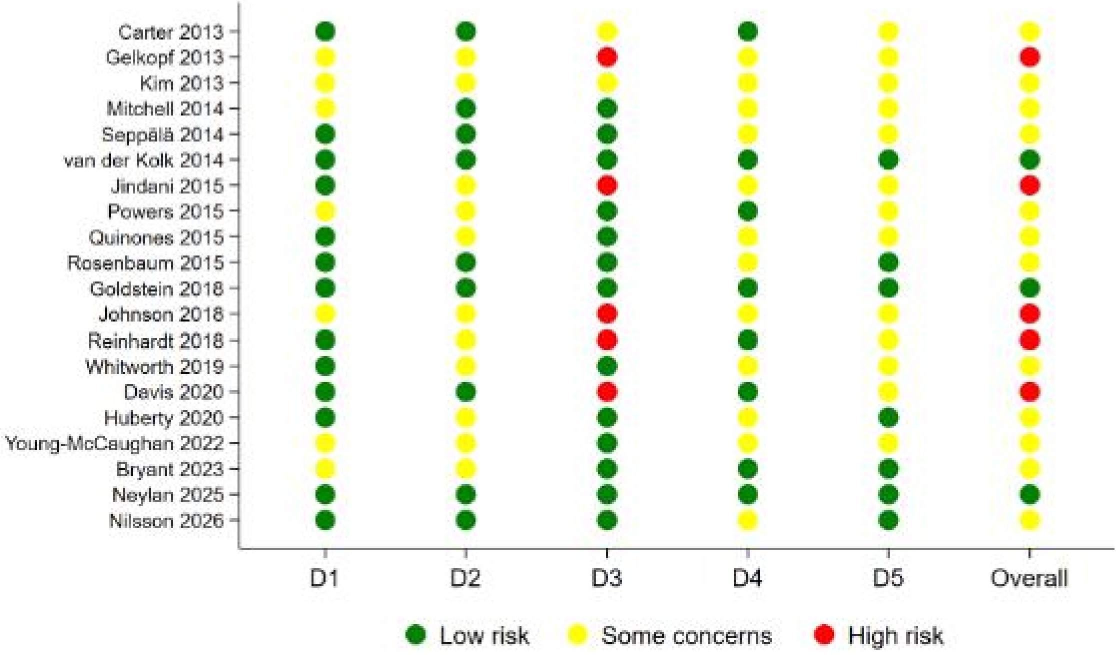

Supplementary Figure S1. Risk-of-bias traffic-light plot for included randomized trials. Risk of bias was assessed using the Cochrane Risk of Bias 2 (RoB 2) tool. Green circles indicate low risk of bias, yellow circles indicate some concerns, and red circles indicate high risk of bias. D1, bias arising from the randomization process; D2, bias due to deviations from intended interventions; D3, bias due to missing outcome data; D4, bias in measurement of the outcome; D5, bias in selection of the reported result.

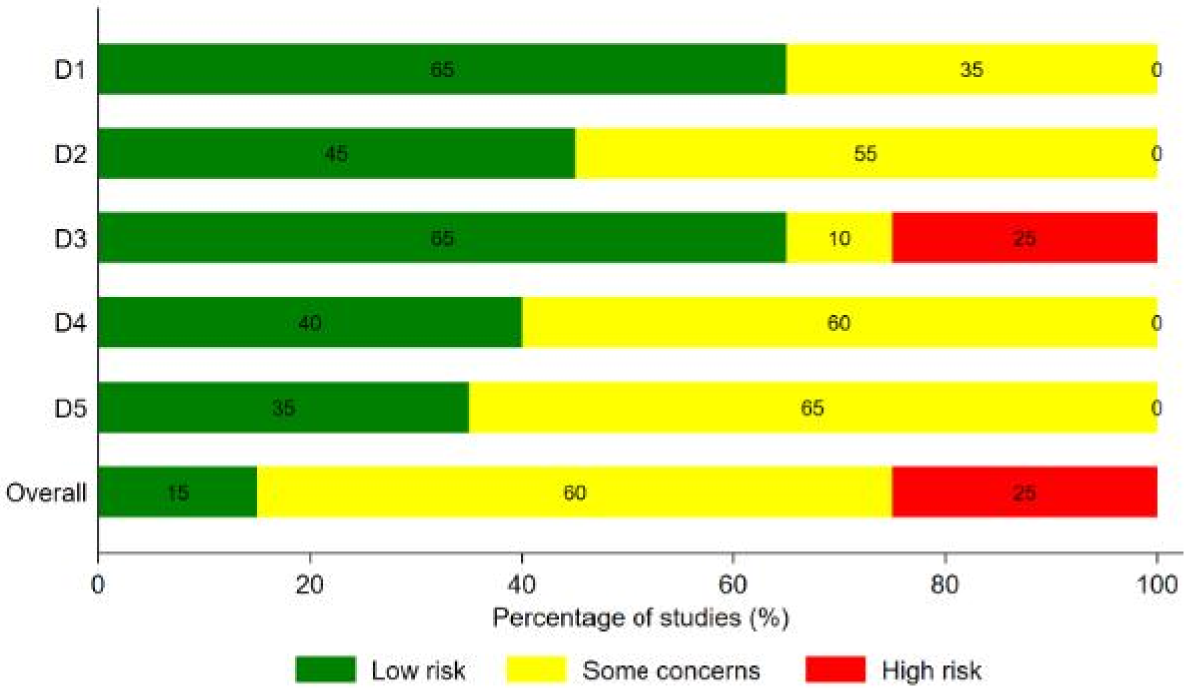

Supplementary Figure S2. Summary of risk-of-bias judgments across included randomized trials. Bars show the proportion of studies rated as low risk of bias, some concerns, or high risk of bias in each domain of the Cochrane Risk of Bias 2 (RoB 2) tool and for the overall judgment. D1, bias arising from the randomization process; D2, bias due to deviations from intended interventions; D3, bias due to missing outcome data; D4, bias in measurement of the outcome; D5, bias in selection of the reported result.

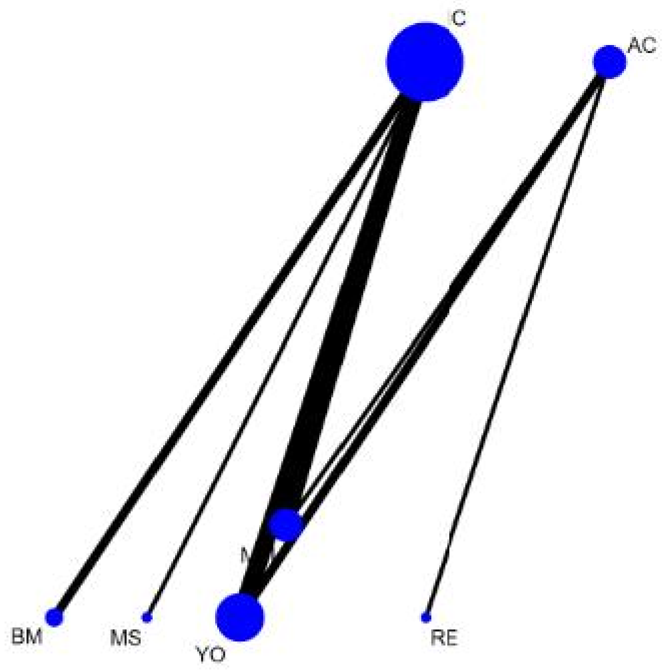

Supplementary Figure S3. Network geometry of the broad exercise and movement-based intervention network. Nodes represent the seven treatment categories included in the broad network, with node size proportional to the number of studies involving each treatment. Edge thickness is proportional to the number of randomized direct comparisons between treatment pairs. The broad network comprised 14 randomized direct comparisons involving 821 participants across seven treatment nodes and extended the strict exercise network by additionally including breathing-meditation and mindful stretching. AC, active control; BM, breathing-meditation; IC, inactive control; MM, multimodal exercise; MS, mindful stretching; RE, resistance exercise; YO, yoga.

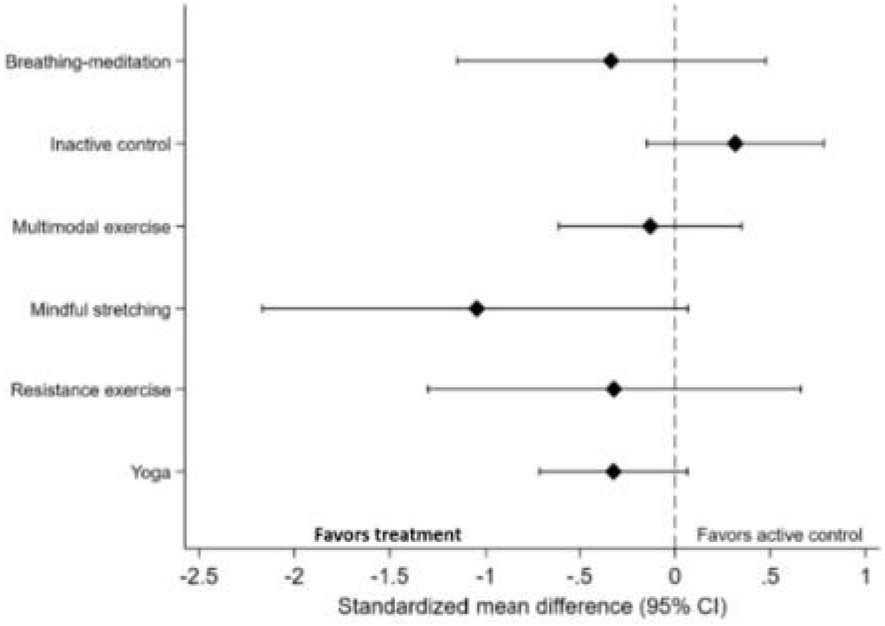

Supplementary Figure S4. Network relative effects in the broad exercise and movement-based intervention network. Treatment effects are presented as standardized mean differences (SMDs) with 95% confidence intervals relative to active control from the random-effects consistency network meta-analysis. Negative values indicate greater reductions in PTSD symptoms relative to active control. The broad network comprised 14 randomized direct comparisons involving 821 participants across seven treatment nodes.

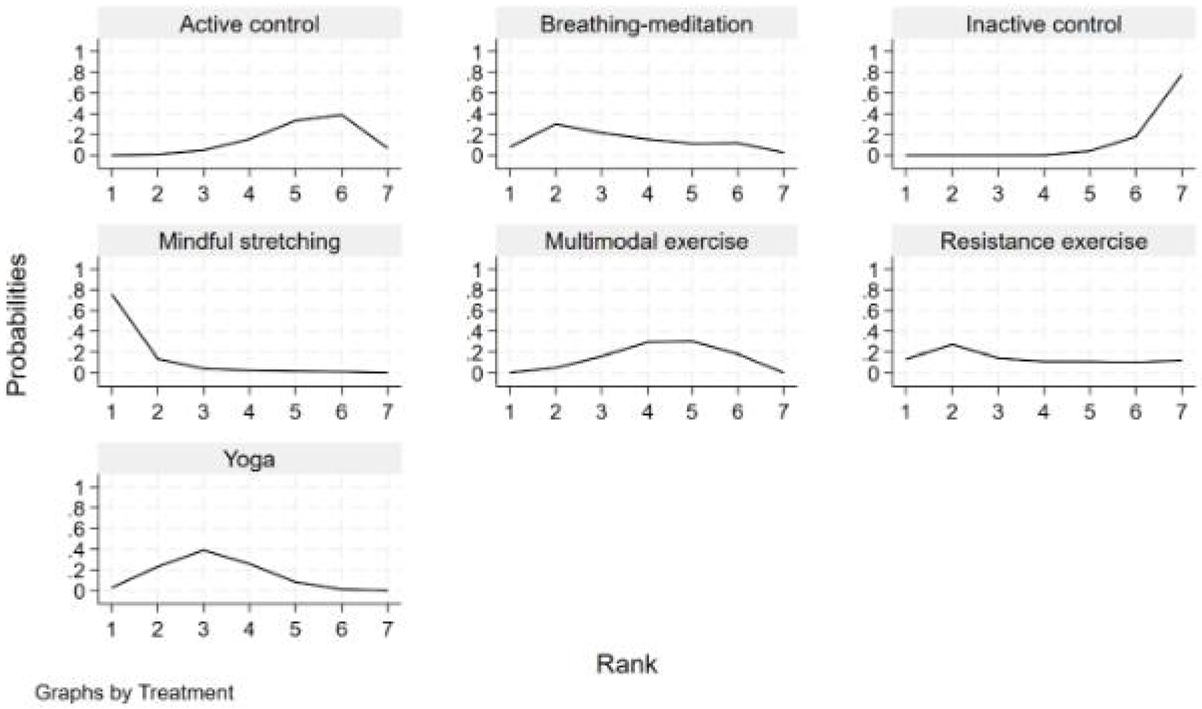

Supplementary Figure S5. Rankograms for treatments in the broad exercise and movement-based intervention network. Each panel shows the probability of a treatment occupying each possible rank, with rank 1 indicating the most favorable treatment. Ranking probabilities were derived from the random-effects consistency model using 10,000 simulation draws (seed = 20260816), with lower PTSD symptom scores indicating more favorable outcomes. The broad network comprised 14 randomized direct comparisons involving 821 participants across seven treatment nodes. Ranking distributions differed from those observed in the strict exercise network.

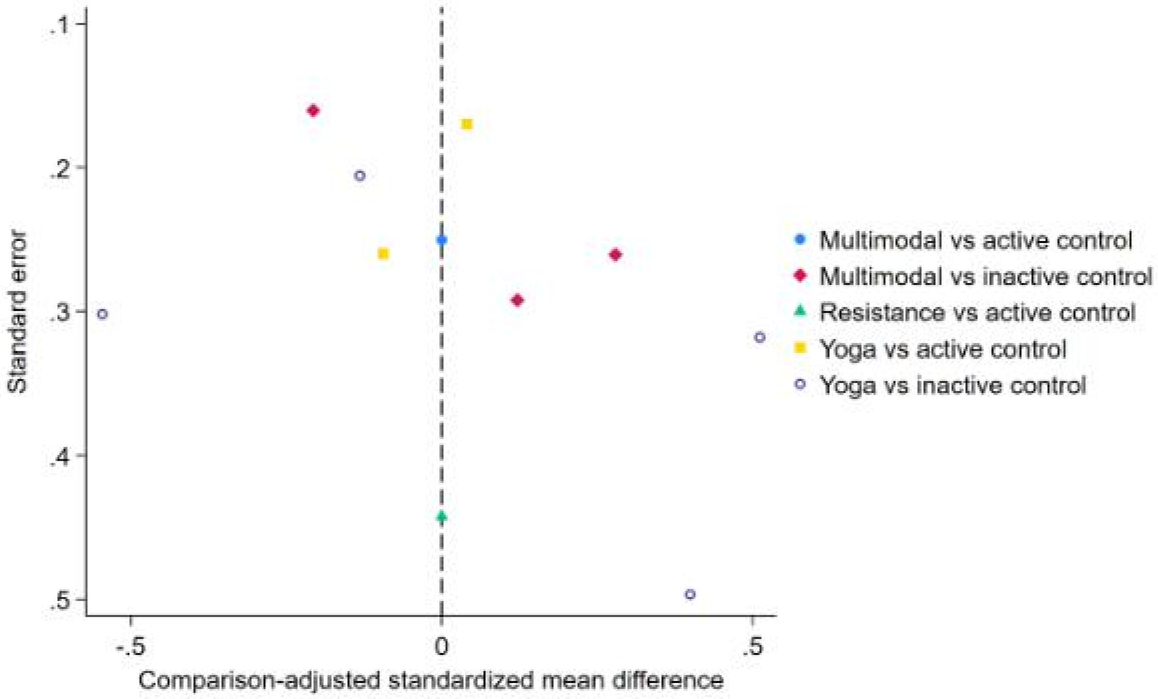

Supplementary Figure S6. Comparison-adjusted funnel plot for the strict exercise network. The horizontal axis represents the difference between each study-specific standardized mean difference and the corresponding random-effects summary estimate for that direct treatment comparison, and the vertical axis represents its standard error. Effects were oriented consistently as exercise intervention versus comparator before centering. Different symbols distinguish the five observed direct treatment comparisons in the strict network. Because the network contained only 11 randomized direct comparisons and several treatment pairs were sparsely informed, the plot was interpreted as an exploratory assessment of small-study effects rather than as definitive evidence of publication bias. AC, active control; IC, inactive control; MM, multimodal exercise; RE, resistance exercise; YO, yoga.

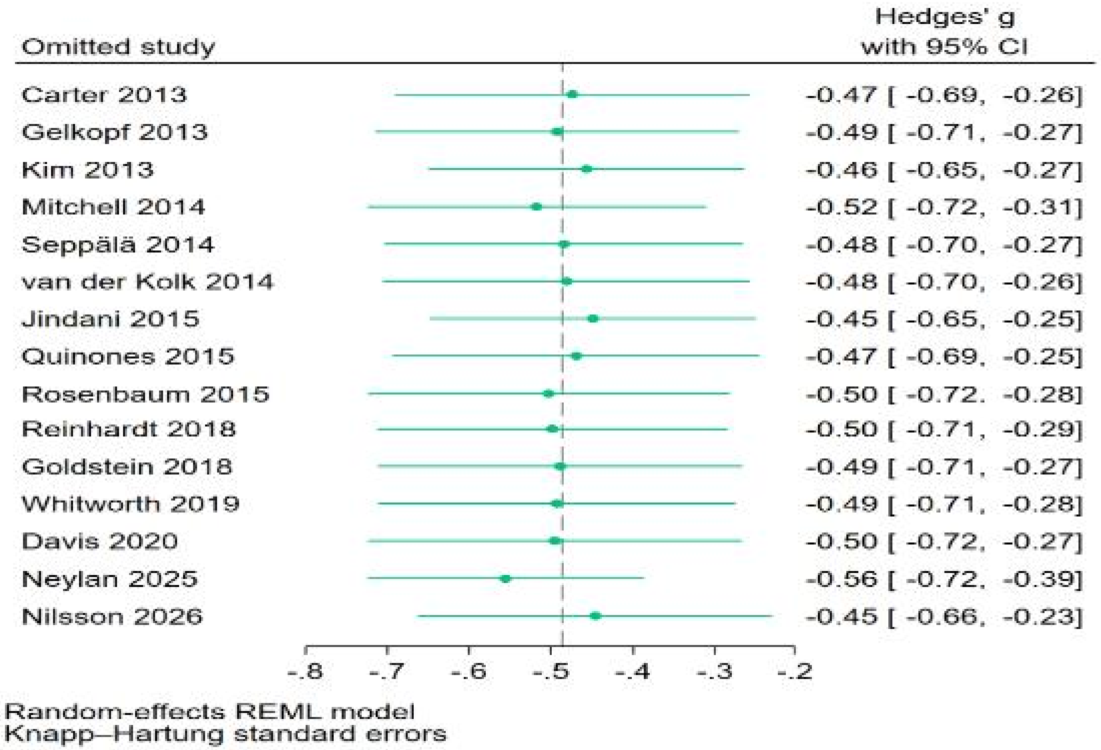

Supplementary Figure S7. Leave-one-study-out sensitivity analysis of the conventional pairwise meta-analysis. Each row presents the pooled Hedges' g and 95% confidence interval obtained after sequentially excluding the indicated study from the 15-study primary pairwise meta-analysis. Estimates were calculated using a random-effects model with restricted maximum likelihood (REML) estimation and Knapp–Hartung adjustment. The vertical dashed line represents the pooled effect estimate from the complete 15-study dataset (Hedges' g= −0.49). Negative values indicate greater reductions in PTSD symptoms in the intervention group relative to the comparator.

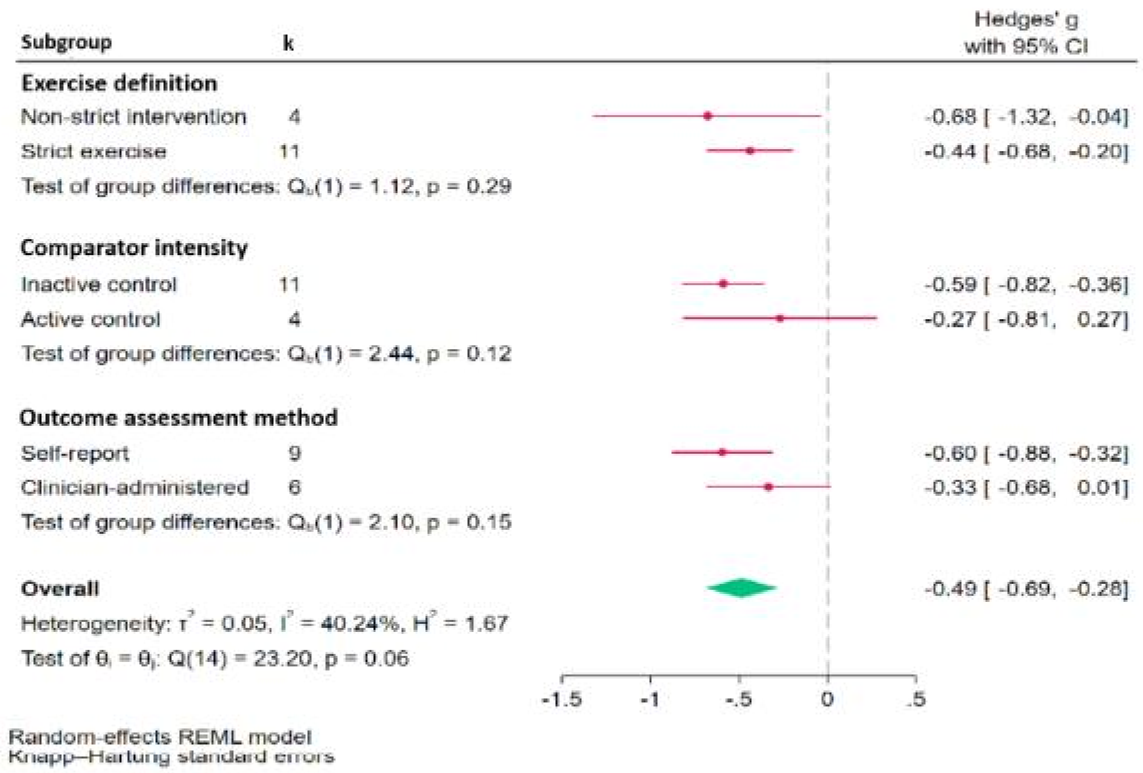

Supplementary Figure S8. Subgroup and sensitivity analyses of the conventional pairwise meta-analysis. Random-effects analyses were conducted according to the strict physical-exercise definition, comparator intensity, and PTSD outcome-assessment method. Pooled effects are presented as Hedges' g with 95% confidence intervals and were estimated using restricted maximum likelihood (REML) with Knapp–Hartung adjustment. Negative values indicate greater reductions in PTSD symptoms in the intervention group relative to the comparator. Tests of between-group differences were interpreted cautiously because several subgroups contained relatively few studies.

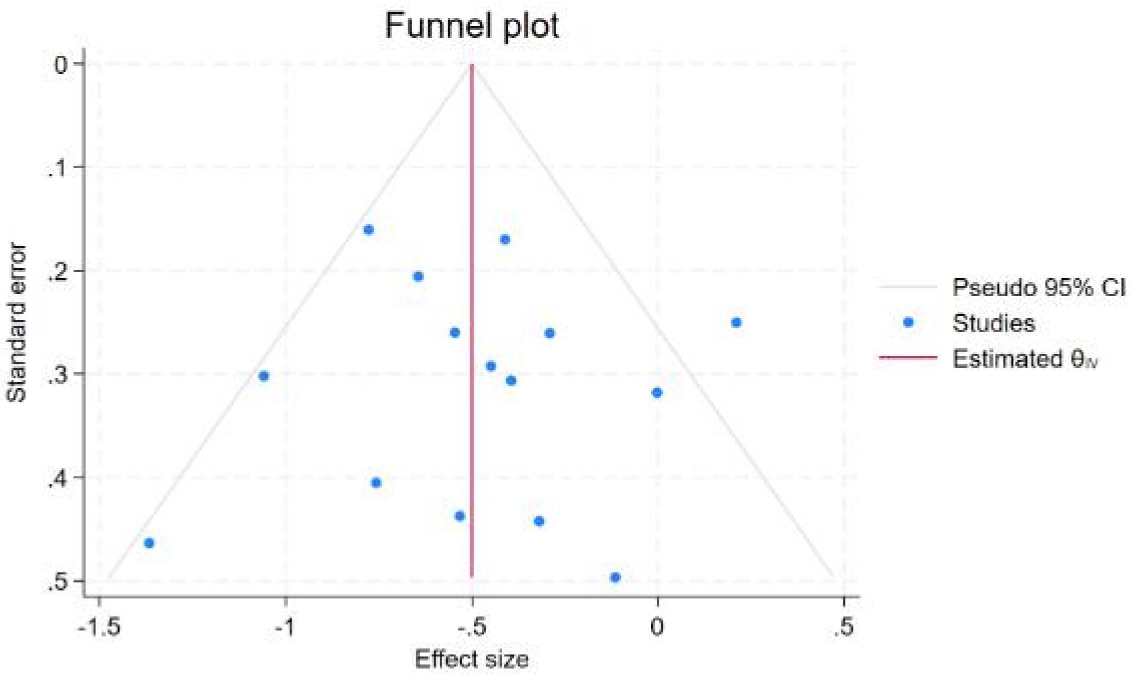

Supplementary Figure S9. Funnel plot for the conventional pairwise meta-analysis. Study-specific Hedges' g values are plotted against their standard errors. The vertical line represents the pooled random-effects estimate, and diagonal lines indicate the pseudo 95% confidence limits. Visual inspection showed no clear evidence of marked funnel-plot asymmetry. Egger's regression test did not indicate statistically significant small-study effects ($\beta = -0.12$, SE = 1.03, $z = -0.11$, $P = .910$). Given the limited number of trials, these findings should be interpreted cautiously and do not exclude the possibility of reporting bias.

Dataset S1: Study-level data and analysis datasets used for the conventional pairwise meta-analysis, strict and broad network meta-analyses, alternative-estimand audit, CINeMA assessment, and risk-of-bias evaluation, including a variable codebook and quality-control checks.
